# Supplementary material for: Fast and interpretable consensus clustering via minipatch learning
Source: PLoS Comput Biol. 2022 Oct 3;18(10):e1010577. doi: 10.1371/journal.pcbi.1010577 (PMC9560608; doi:10.1371/journal.pcbi.1010577)
Supplement: S1 Text — (DOCX) [file pcbi.1010577.s001.docx]

FAST AND INTERPRETABLE CONSENSUS CLUSTERING VIA MINIPATCH LEARNING:

SUPPLEMENTARY MATERIALS

A PREPRINT

Luqin Gan ^∗^ Genevera I. Allen ^†, ‡^

July 24, 2022

# A Propositions

A.1 Proporsition 1

Proposition 1. *The computational complexity of MPCC in Algorithm 1 is O*(*mn*^2^*T* +*N*^2^)*, where T is the total number of minipatches.*

*Proof.* Firstly, construction of minipatch requires *O*(1) time complexity. Then we need
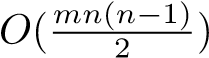
 to calculate the distance matrix of minipatch and *O*(*n*^2^) to perform hierarchical clustering with Ward.D linkage [Murtagh,1983].

The time complexity is
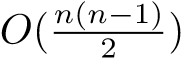
 for both co-clustering membership matrix *V* and co-sampling matrix *D* updates. Finally, after *T* iterations, when we calculate the consensus matrix *S*, *O*(*N*^2^) operations are needed. Therefore, the overall time complexity over *T* iterations is *O*((*mn*(*n* − 1) + *n*^2^ + *n*(*n* − 1)) × *T* + *N*^2^), which can be simplified to *O*(*mn*^2^*T* + *N*^2^).

A.2 Proporsition 2

The following result probabilistically bounds the deviations of the distances computed using only a subset of features:

Proposition 2. *For ϵ >* 0 *and*
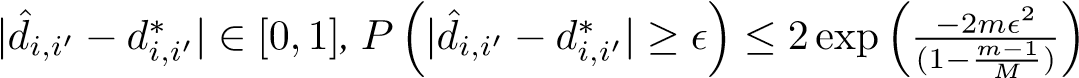
*,*

where
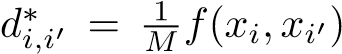
 is the distance between observations *x_i_* and *x_i_*′ using the full set of *M* features, and
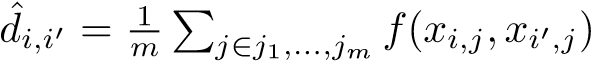
 is the distance using a subset of *m* features. This is derived from the Hoeffding

inequality [46]. This result states that the probability that distances computed on minipatches are far off from original distances is small, under the worst-case scenario.

# B Early Stopping Criteria

To avoid unnecessary iterations in an effort to optimize computational efficiency, we employ an early stopping criteria which stops the algorithm once the consensus matrix is stable. The stability can be measured by the changes in confusion values [Ren et al., 2017], where the confusion is defined as


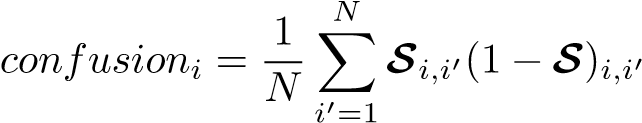


∗

Department of Statistics, Rice University, Houston, Texas 77005

†

Departments of Electrical and Computer Engineering, Statistics, and Computer Science, Rice University, Houston, Texas, 77005

‡

Duncan Neurological Research Institute, Baylor College of Medicine, Houston, Texas, 77030

for observation *i*. We consider the consensus results to be stable if the confusion values have no or little changes over several iterations. Let
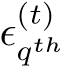
 be the absolute difference between the *q^th^* percentile of confusion values at iteration *t* and *t* − 1; and Ω*_q,c_* denotes the number of times that *ϵ_q_th* is continuously less than a constant *c*. We propose to stop the algorithm when the 90% percentile value of the confusions remains small and unchanged for the past 5 iterations, that is, Ω_90_*_,_*_0_*_._*_00001_ *<* 5 for *c* = 0*.*00001. Such stopping criteria is used in our empirical study and proved to achieve a great balance between clustering accuracy and computational efficiency.

# C Benchmarking

In this section, we include the detailed implementation of competing methods in the study of synthetic data and real data. For all methods, we use the oracle number of clusters *K*.

- IMPACC and MPCC: we use default hyper-parameters and apply the early-stopping rule;
- Consensus clustering: the number of observations to subsample and number of iterations is set to be the same as MPCC with an early-stopping rule;
- sparse KMeans: we first tune hyper-parameters with the provided permutation function, setting the range of parameters [3,7]; then, we apply sparse KMeans clustering with the selected optimal hyper-parameter and oracle number of clusters.
- sparse hierarchical clustering: we first tune hyper-parameters with the provided permutation function, setting the range of parameters to be [1.5,6]; then we apply sparse hierarchical clustering with the selected optimal hyper-parameter and average linkage;
- Seurat: we conduct the following steps to perform Seurat clustering:
  1. Using the count data without log transformation, we first find a subset of 2000 features that exhibit high cell-to-cell variation using *vst* method;
  2. Then, we scale the selected subset of data and apply PCA dimension reduction;
  3. Using the first 50 PCs, we group the observations with the resolution value that can result in the oracle number *K* of clusters.

Note that we only apply Seurat to single-cell data sets.

- SC3: we first create a SingleCellExperiment object with count and log-transformed data; then perform SC3 with the number of clusters being oracle and other hyper-parameters being the default. Note that we only apply SC3 to single-cell data sets;
- KMeans: we apply KMeans to the scaled and log-transformed data with oracle number of clusters;
- Hierarchical clustering: we apply hierarchical clustering to the scaled and log-transformed data with oracle number of clusters, using ward.D linkage with Manhattan distance;
- Spectral clustering: we apply spectral clustering to the scaled and log-transformed data with oracle number of clusters, using rbfdot kernel;
- KMedoid clustering: we apply KMedoid clustering to the scaled and log-transformed data with the oracle number of clusters;
- Clusterings after t-SNE dimension reduction: we first apply t-SNE dimension reduction to the scaled and log-transformed data, with perplexity being 30; then we apply KMeans/ Hierarchical clustering/ Spectral clustering/ KMedoid clustering to the reduced dimensions, with settings the same as above.

# D Additional Study on Synthetic Data

D.1 Sparse Block-Diagonal Simulation

In the sparse block-diagonal simulation study, each data set is created from a mixture of Gaussian with block-diagonal covariance matrix
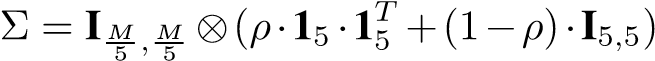
, where ⊗ denotes the Kronecker product. The parameter *ρ* is set to be 0*.*5. With other settings being the same as the sparse autoregressive simulation in Section 3.1, we compare the model performance of IMPACC and MPCC to other competing methods. As shown in Figure 5, the results of the sparse block-diagonal scenario are quite similar to that in sparse autoregressive simulation, with IMPACC outperforming all other methods in terms of clustering accuracy, measured by ARI. Also, IMPACC demonstrates better feature selection accuracy measured by the F1 score and much fewer computation costs than other sparse clustering methods.


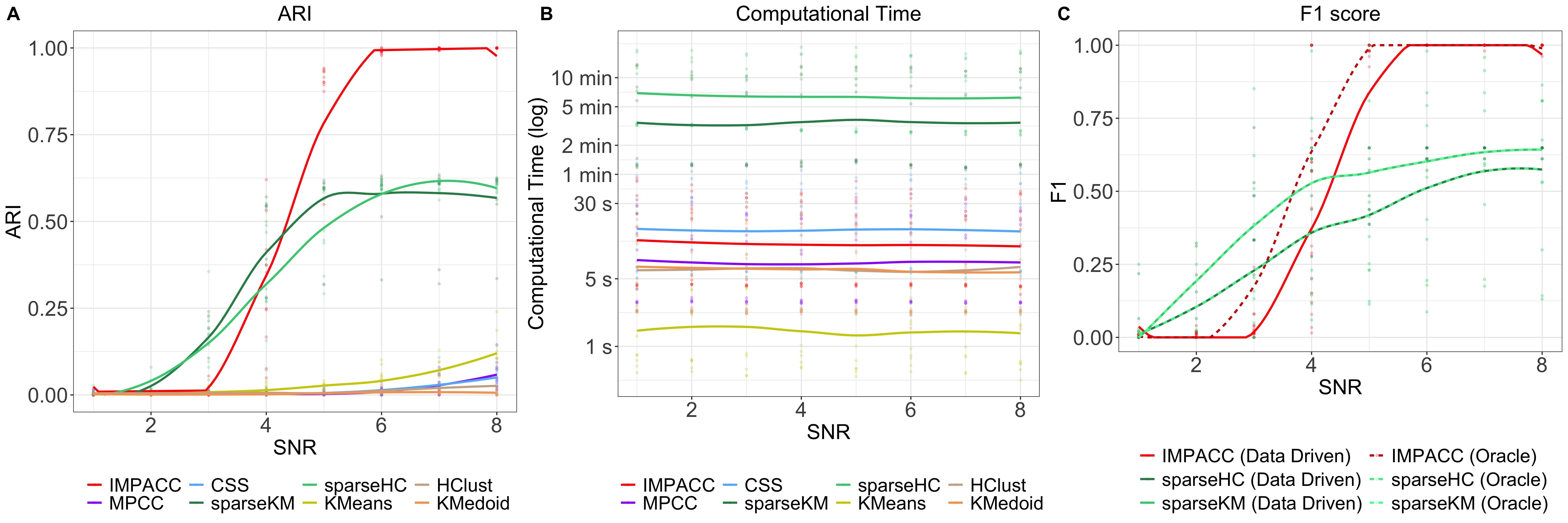


Figure 5: Clustering performance (ARI), feature selection accuracy (F1 score), and computation time on sparse synthetic data sets. (A) ARI (higher is better) of estimated grouping; (B) computation time in log seconds; (C) F1 score for signal feature estimates. IMPACC has superior performance over competing methods in clustering and feature selection accuracy with significant computational savings.

D.2 Weak Sparse Block-Diagonal Simulation

In order to further investigate the effectiveness of MPCC and IMPACC methods, we propose to evaluate the methods on more noisy and challenging data sets. To do so, we construct a weak sparse scenario, in which we generate noise features with higher variance than that of sparse block-diagonal simulation as in Appendix D.1. Instead of setting the means of all noise features to be 0, here, we design the weak sparse simulation with means of noise features *µ*_0_ ∼ *N*(0_4975_*,I*_4975_),

where *I* is the identity matrix. With other settings being exactly the same as the sparse block-diagonal simulation in Appendix D.1, we compare the model performance of IMPACC and MPCC to other competing methods. As shown in Figure 6, the results of the weak sparse scenario are pretty similar to that in sparse simulation, with IMPACC outperforming all other methods in terms of clustering accuracy, measured by ARI. Also, IMPACC demonstrates better feature selection accuracy measured by the F1 score and much fewer computation costs than other sparse clustering methods.


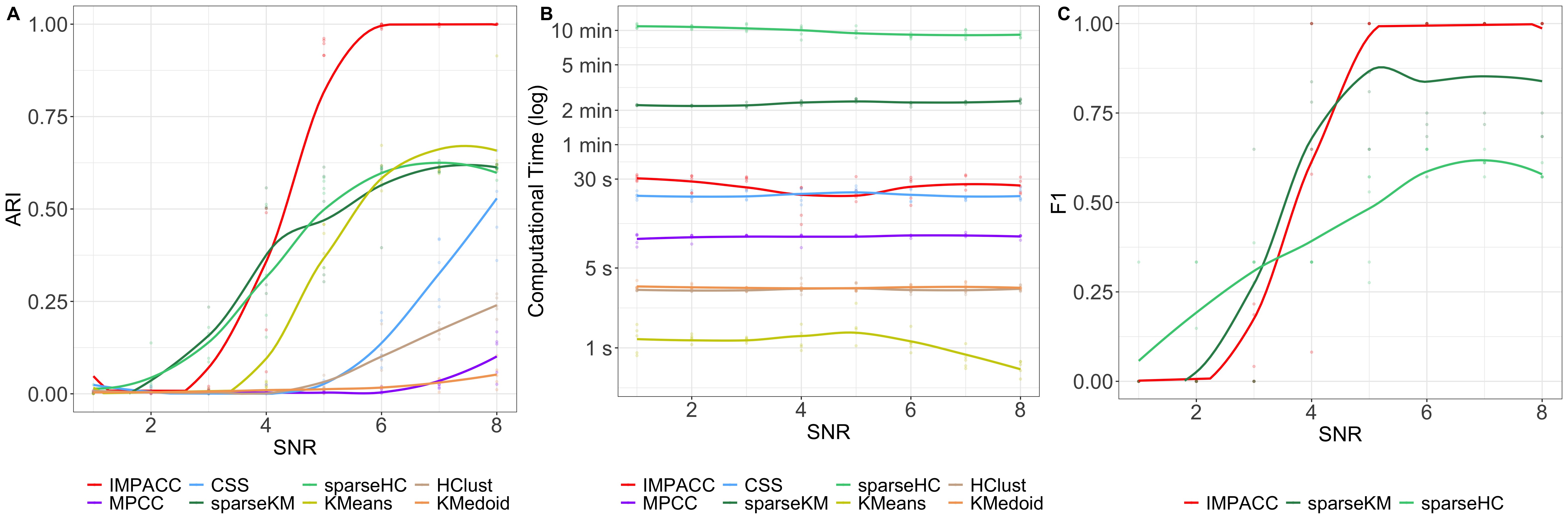


Figure 6: Clustering performance (ARI), feature selection accuracy (F1 score), and computation time on weak sparse synthetic data sets. (A) ARI (higher is better) of estimated grouping; (B) computation time in log seconds; (C) F1 score for signal feature estimates. The performance is quite similar to sparse simulation that our method IMPACC has superior performance over competing methods in terms of clustering performance and similar feature selection accuracy

with significant improvement on computational cost.

D.3 No Sparse Block-Diagonal Simulation

As our previous synthetic data sets are built with sparse and high dimensional settings, one may ask how our methods perform in the case of no sparsity and low dimensions? For comparison, we generate low dimensional data sets where the number of features (*M* = 100) is smaller than the number of observations(*N* = 500), with all the features relevant to


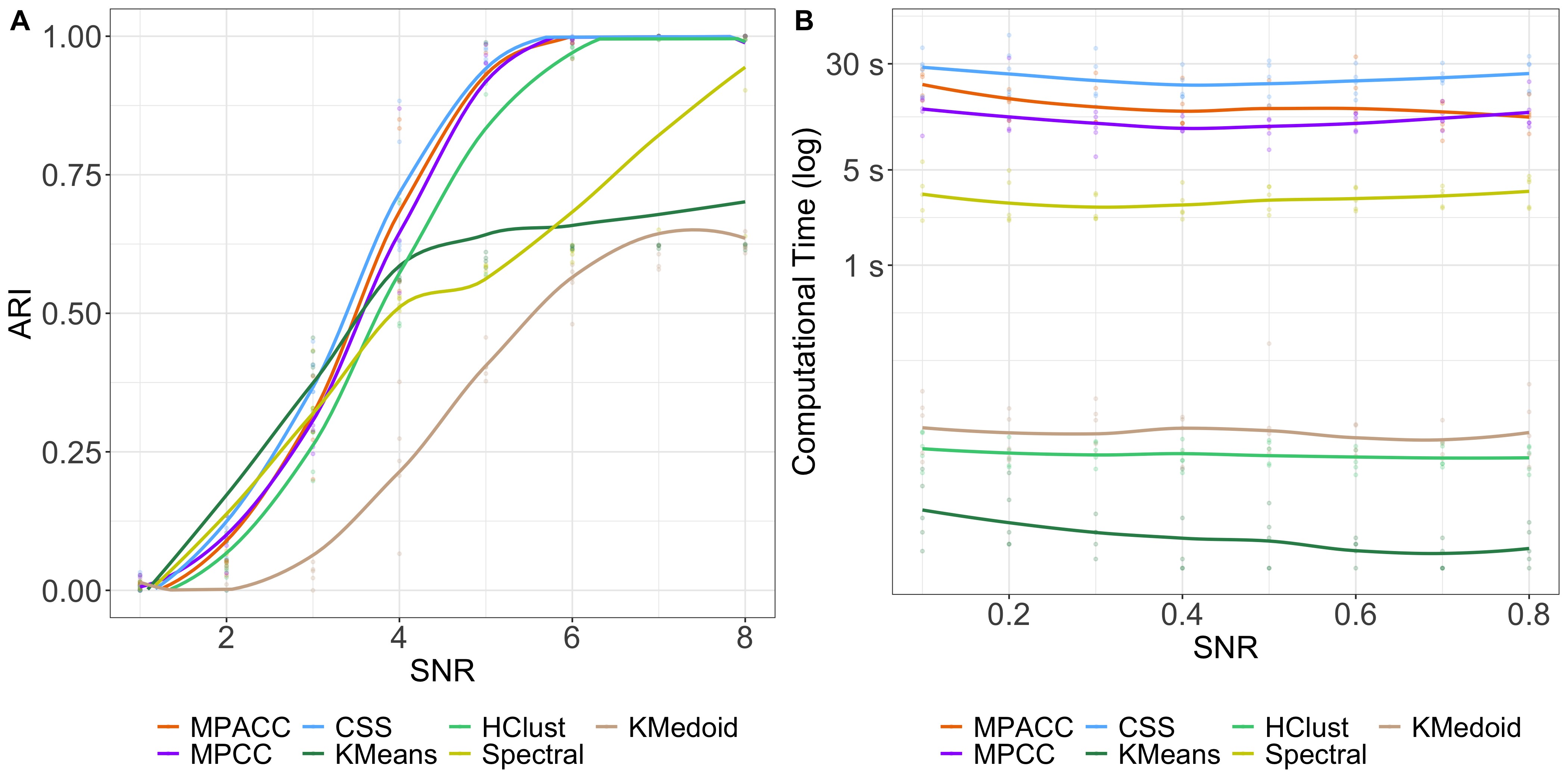


Figure 7: Clustering performance (ARI) and computation time on no sparsity and low dimensional synthetic data sets.

(A) Adjusted rand index (higher is better) of estimated grouping; (B) computation time in log seconds; Our method MPACC and MPCC have similar clustering accuracy and advantages in computation time compared to consensus clustering. They also demonstrate much higher ARI over other competing methods.

differentiate the clusters. Similar to the sparse scenarios, the no sparse data sets are simulated from a mixture of Gaussian with block-diagonal covariance matrix. Differently, the number of features is set to be *M* = 100, and other values are the same as in sparse simulation. Without any noise feature, the 100 signal features are generated with *µ* = [*µ_k_*], such that *µ_k_* ∼ *N*(*u_k_,*0*.*1), and we set
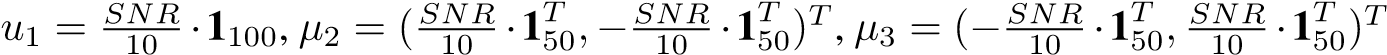
,


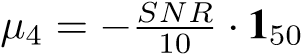
. In this case, all features are important in discovering clusters, so we do not implement clustering

algorithms with feature selection, including IMPACC, sparseKM, and sparseHC. We aim to compare MPACC, which only adaptively subsamples observations, and MPCC, which uses a random sampling scheme, to consensus clustering and other widely used algorithms. As shown in Figure 7, we can tell that MPACC and MPCC do not offer significant improvements in terms of clustering accuracy, compared to consensus clustering in the no sparsity setting. But our methods show better computational efficiency than consensus clustering. Besides, MPACC and MPCC still result in much better clustering accuracy than other standard clustering algorithms, including K-Means, K-Medoid, and spectral clustering.

D.4 Additional Results on Sparse Autoregressive Simulation

One may ask can we implement algorithms other than hierarchical clustering on the consensus matrix in the final step, and whether the methods would perform differently? In practice, our frameworks are quite flexible. We can implement any other clustering algorithms in the final step, as long as they rely on a distance matrix or similarity matrix of the data. For example, we can apply hierarchical clustering or K-Medoid clustering with 1 − *S* as a distance matrix, or spectral clustering using *S* as a similarity matrix. We compare our frameworks’ clustering accuracy to existing methods in the sparse simulation scenario setting, adding spectral clustering as the final algorithm in IMPACC, MPCC, and consensus clustering. As shown in Figure 8, the methods using hierarchical and spectral clustering as the final algorithm are indicated by *(HC)* and *(Spec)*, respectively. Since the only difference is the final clustering applied to the consensus matrix, only the clustering accuracy would be influenced. IMPACC with spectral clustering as the final algorithm *IMAPCC(Spec)* has almost the same ARI as that of using hierarchical clustering as the final algorithm *IMAPCC(HC)*. And in both MPCC and consensus clustering, spectral clustering as the final algorithm significantly enhances clustering accuracy. Therefore, our approaches are flexible and widely applicable to numerous different models in the choices of final clustering algorithms so that users can optimize model settings with respect to data of interest.

D.5 Splatter Synthetic Data Based on Goolam

Splatter[1] single-cell simulation generates synthetic data based on a gamma-Poisson distribution, with parameters estimated from a real single-cell data set. Here we generate parameters from the Goolam single-cell

RNA-seq data [2], and we set the number of observations, features and clusters to be *N* = 500, *M* = 5*,*000, *K* = 3, respectively. We design unbalanced cluster sizes with the percentage of observations in each cluster to be 15%, 25%, 60%, and the probability that a gene will be selected to be differentially expressed as 0*.*03. In addition, the difficulty of clustering can be controlled by the differential expression factors, which are produced from a


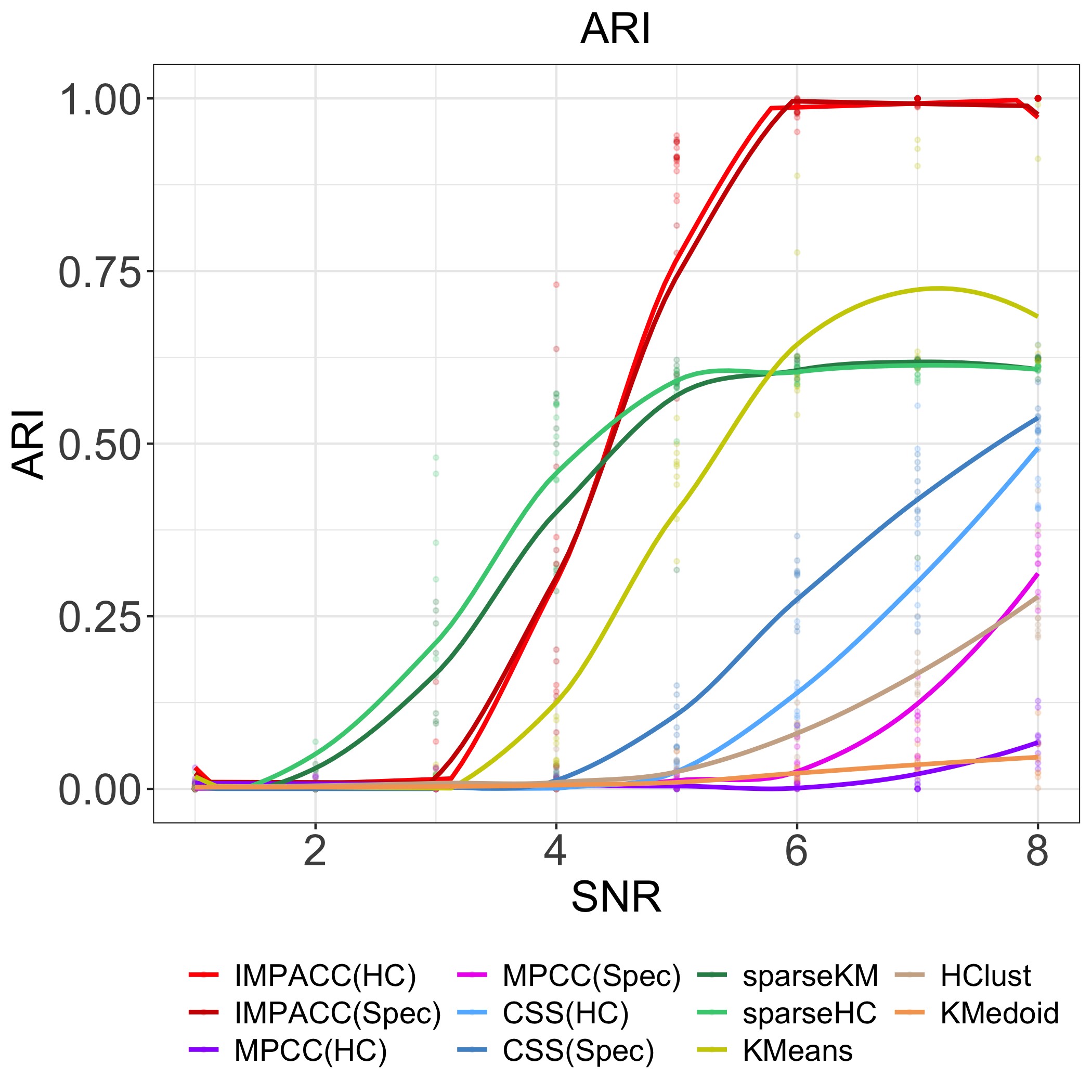


Figure 8: Clustering performance (ARI) on sparse data sets autoregressive covariance structure, adding results of spectral clustering as the final algorithm. Methods using hierarchical and spectral clustering are indicated by (HC) and (Spec), respectively. IMPACC has similar ARI in both *HC* and *Spec* settings, while MPCC and consensus clustering with *Spec* show better clustering accuracy than that with *HC* setting.

log-normal distribution. Here we set the scale parameter to be 0*.*3 and the location parameter to be *SNR/*10, with *SNR* ranging from 1 to 8.

Here we also compare the SC3 method, which can conduct feature selection with the adjusted p-value for differential genes. We can select the top oracle number of features with the smallest adjusted p-values, or all features with p-values smaller than 0*.*05 in a data-driven way. Note that the splatter-simulated data set is a difficult task for analyzing differentially expressed genes, so the F1 score for all the feature selection methods is quite low, and IMPACC can have relatively better performance than other competitors. IMPACC still has superior performance in clustering accuracy and less computational burden than other sparse methods.


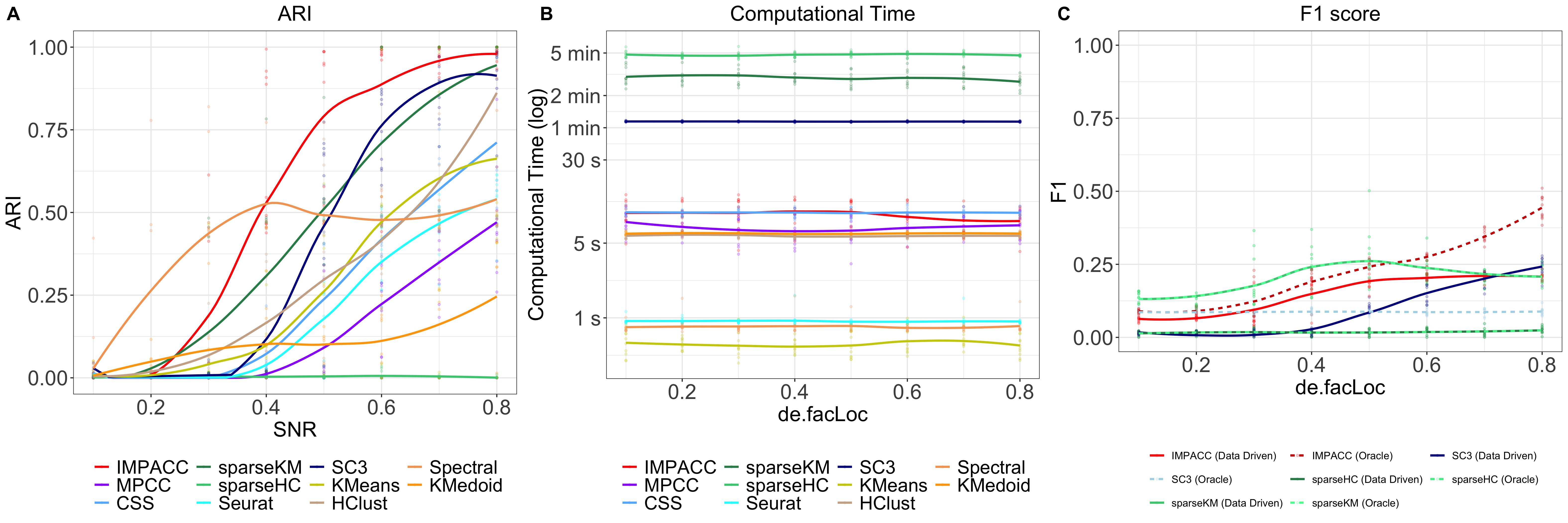


Figure 9: Clustering performance (ARI), feature selection accuracy (F1 score), and computation time on splatter synthetic data sets. (A) ARI (higher is better) of estimated grouping; (B) computation time in log seconds; (C) F1 score for signal feature estimates. IMPACC still has superior performance over competing methods in clustering accuracy

with significant computational savings. Even though the feature selection accuracy is low for all the methods, IMPACC can have relatively better performance than SC3 and sparseHC.


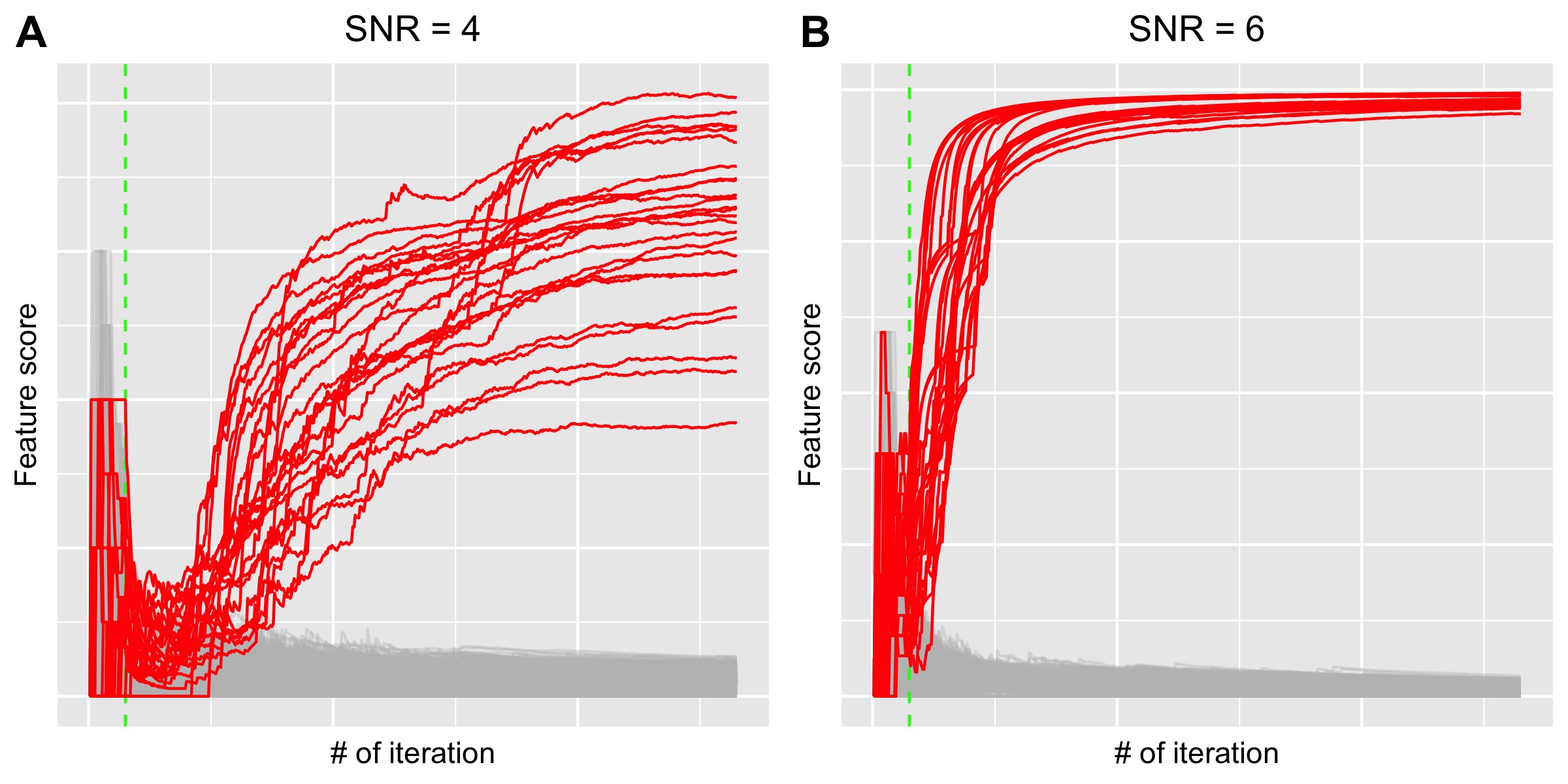


Figure 10: Feature scores of IMAPCC versus number of iterations in the sparse scenario with *SNR* = 4 and *SNR* = 6. Our method is able to distinguish signal features from the noise, with speed of convergence and level of score separation relative to the difficulty of the data sets.

D.6 Patterns of Feature Scores

One may also be interested in the pattern of feature scores over iterations. We display feature scores obtained from IMPACC versus the number of iterations in the sparse block-diagonal synthetic data study, in the case of *SNR* = 4 and *SNR* = 6. From Figure 10, we see that IMPACC can correctly identify all the signal features and noise features after the burn-in stage. In addition, the feature scores converge faster and have a clearer separation between the important and noise features in the data with higher *SNR*. It is reasonable because the important features in such data have stronger signals, so it is easier for us to separate the relevant features from the noisy ones and thus easier to cluster the observations.

# E GO Enrichment Analysis on Yan Data Set

The GO enrichment analysis for EPI-specific genes compared to all the other cells after the 8-cell stage, reported in Yan et al. [3]:

- transcription
- regulation of transcription
- regulation of transcription,
- DNA-dependent regulation of RNA metabolic process
- sexual reproduction
- gamete generation
- reproductive cellular process
- transcription initiation from RNA polymerase II promoter
- spermatogenesis
- male gamete generation
- germ cell development
- multicellular organism reproduction
- reproductive process in a multicellular organism
- RNA biosynthetic process
- transcription, DNA-dependent
- transcription from RNA polymerase II promoter
- transcription initiation
- reproductive developmental process
- nucleosome assembly
- chromatin assembly
- regulation of chromosome organization

We identify genes with adjusted p-values less than 0.05 in differential tests from SC3 as differentially expressed genes and with non-zero feature weights in spraseKM as differentially expressed genes. Table 1 and Table 2 demonstrate the GO enrichment analysis based on IMPACC and sparseKM, respectively, and the results using SC3 can be found in Table S2.

Table 1: IMPACC GO enriched pathway analysis on YAN data.

| Pathway | p-value | Gene Ratio | Bg Ratio | Count |
| --- | --- | --- | --- | --- |
| oocyte differentiation | 0.001 | 9/363 | 47/18862 | 9 |
| regulation of reproductive process | 0.001 | 15/363 | 159/18862 | 15 |
| DNA methylation involved in gamete generation | 0.00132 | 6/363 | 19/18862 | 6 |
| oocyte development | 0.00132 | 8/363 | 43/18862 | 8 |
| female gamete generation | 0.00132 | 13/363 | 131/18862 | 13 |
| oogenesis | 0.00392 | 10/363 | 85/18862 | 10 |
| cellular process involved in reproduction in multicellular organism | 0.00399 | 21/363 | 357/18862 | 21 |
| negative regulation of reproductive process | 0.00695 | 8/363 | 57/18862 | 8 |
| positive regulation of reproductive process | 0.01115 | 9/363 | 80/18862 | 9 |
| negative regulation of binding of sperm to zona pellucida | 0.01225 | 3/363 | 4/18862 | 3 |
| regulation of binding of sperm to zona pellucida | 0.02745 | 3/363 | 5/18862 | 3 |
| regulation of action potential | 0.03099 | 7/363 | 55/18862 | 7 |
| egg coat | 0.00028 | 4/370 | 5/19520 | 4 |
| presynapse | 0.00487 | 24/370 | 487/19520 | 24 |
| pi-body | 0.02793 | 3/370 | 7/19520 | 3 |
| exocytic vesicle | 0.02793 | 13/370 | 215/19520 | 13 |
| basement membrane | 0.03782 | 8/370 | 94/19520 | 8 |
| pole plasm | 0.04455 | 4/370 | 21/19520 | 4 |
| ammonia-lyase activity | 0.02692 | 3/368 | 5/18337 | 3 |
| acrosin binding | 0.02692 | 3/368 | 5/18337 | 3 |
| calcium-dependent phospholipid binding | 0.02692 | 7/368 | 54/18337 | 7 |
| phospholipase A2 inhibitor activity | 0.03177 | 3/368 | 6/18337 | 3 |
| aspartic-type endopeptidase inhibitor activity | 0.04162 | 3/368 | 7/18337 | 3 |
| structural constituent of egg coat | 0.04162 | 2/368 | 2/18337 | 2 |
| hepoxilin A3 synthase activity | 0.04162 | 2/368 | 2/18337 | 2 |
| oleate transmembrane transporter activity | 0.04162 | 2/368 | 2/18337 | 2 |

Table 2: sparseKM GO enriched pathway analysis on YAN data.

| Pathway | p-value | Gene Ratio | Bg Ratio | Count |
| --- | --- | --- | --- | --- |
| oocyte differentiation | 0.00125 | 5/70 | 47/18862 | 5 |
| female gamete generation | 0.00688 | 6/70 | 131/18862 | 6 |
| oogenesis | 0.00726 | 5/70 | 85/18862 | 5 |
| oocyte development | 0.00726 | 4/70 | 43/18862 | 4 |
| forebrain neuroblast division | 0.02448 | 2/70 | 4/18862 | 2 |
| gonad development | 0.03314 | 6/70 | 212/18862 | 6 |
| development of primary sexual characteristics | 0.03314 | 6/70 | 217/18862 | 6 |
| egg coat | 0.01675 | 2/68 | 5/19520 | 2 |
| structural constituent of egg coat | 0.00332 | 2/66 | 2/18337 | 2 |
| acrosin binding | 0.01647 | 2/66 | 5/18337 | 2 |

# F Dimension Reduction & visualization

Informative dimension reduction and visualization can be achieved by applying multidimensional scaling to the distance matrix obtained from IMPACC, denoted as MDS-IMPACC. The embedding is produced by implementing monotone spline transformed MDS to 1−*S*, which can be regarded as an interpretable distance matrix estimation of the data. Both the number of knots and spline degree are set to be 2 by default. MDS-IMPACC has significantly better visualization quality than commonly used dimension reduction techniques, including PCA, UMAP, and t-SNE. The visualization produced by MDS-IMPACC is equipped with strong interpretability by preserving pairwise distance structure using multidimensional scaling. Moreover, MDS-IMPACC is possible to provide additional information on the levels of clustering uncertainty of the observations, using confusions derived from the final consensus matrix of IMPACC. Figure 12 shows the results of applying MDS-IMPACC, PCA, t-SNE, and UMAP in the bulk-cell RNA-seq and


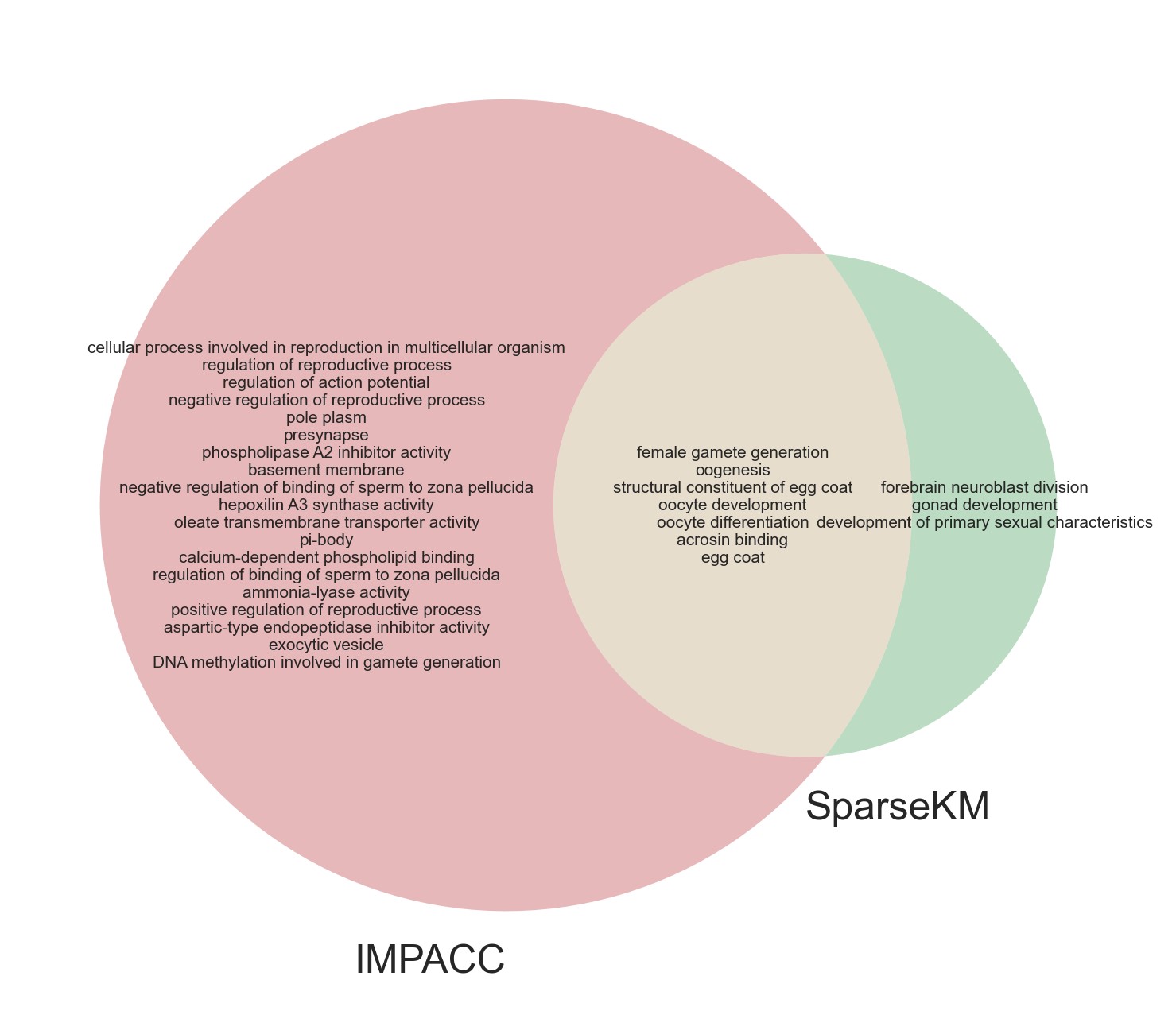


Figure 11: GO terms identified by IMPACC and sparseKM.

single-cell RNA-seq data sets. MDS-IMPACC can yield scatterplots with tighter groups of cells and clearer separations among different types of cells than PCA and t-SNE. Therefore, based on monotone spline multidimensional scaling, IMPACC is able to provide a new interpretable and informative dimension reduction and visualization.


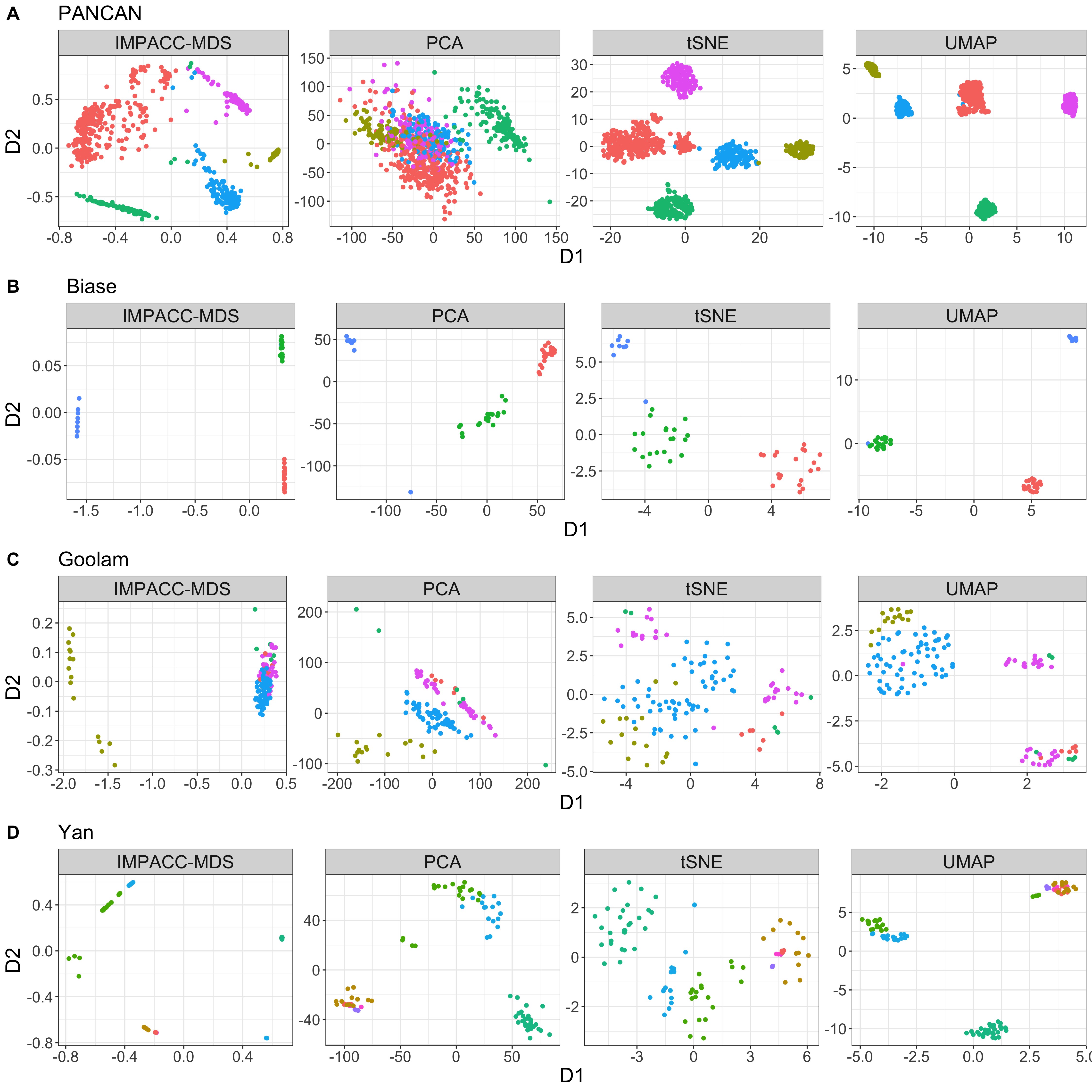


Figure 12: Dimension reduction accuracy. Scatterplots of the top two dimension of the cells colored by cell type, derived from MDS-IMPACC, PCA, t-SNE and UMAP. Scatterplots from IMPACC-MDS can yield concentrated clusters

with clearer boundaries.

# G Comparison of IMPACC variants

We constructed variants of IMPACC by replacing simple methodologies with more complex ones. Specifically, we focus on the following two aspects.

1. How to evaluate association between features and cluster labels in a minipatch. Our current IMPACC evaluates the level of association between each feature and the estimated cluster labels on that minipatch by a one-way ANOVA test. We first consider a more complex alternative: a non-parametric ANOVA (Kruskal-Wallis test), which relaxes normality assumptions. Secondly, we agree with reviewer 2 that the ANOVA test does not consider feature collinearity. Therefore, we propose to conduct a multinomial regression of features to predict cluster assignments, which can account for feature correlations. Specifically, one feature’s association level is calculated as the mean of predicted probabilities on the cluster assignments from the fitted multinomial regression.
2. How to determine the number of clusters *k* in a minipatch. Our current IMPACC method cuts the hierarchical tree at the *h* quantile (typically set to be 0.95). We consider two alternatives: 1). for a given range of *k* values,

we select the one that can generate the highest silhouette score; 2). use the oracle number of clusters *K* as in regular consensus clustering and SC3.

With other parts of the algorithm being the same, we apply the proposed variants to the three benchmark single-cell RNA-seq data. As shown in Figure G, IMPACC variants that implement rank ANOVA test or multinomial regression do not demonstrate advantages in clustering accuracy, measured by ARI. Also, the variants have a more significant computational burden than the IMPACC with a simple ANOVA test.

In addition, Figure G compares different alternatives to determine the number of clusters in a minipatch. The IMPACC variants can have slightly higher ARI in some cases, but can not outperform IMPACC, which cut the hierarchical tree at a default 0*.*95 quantile, in clustering accuracy and computational time simultaneously. Therefore, we can conclude that even though IMPACC implements simple procedures in the algorithm, it does compromise accuracy for computational efficiency. Some may wonder why the variants with oracle *K* have greater computational time in some data sets. It is because we implement early stopping criteria based on the changes of the consensus matrix, and the variant with oracle *K* would take more iterations to reach a stable consensus matrix.


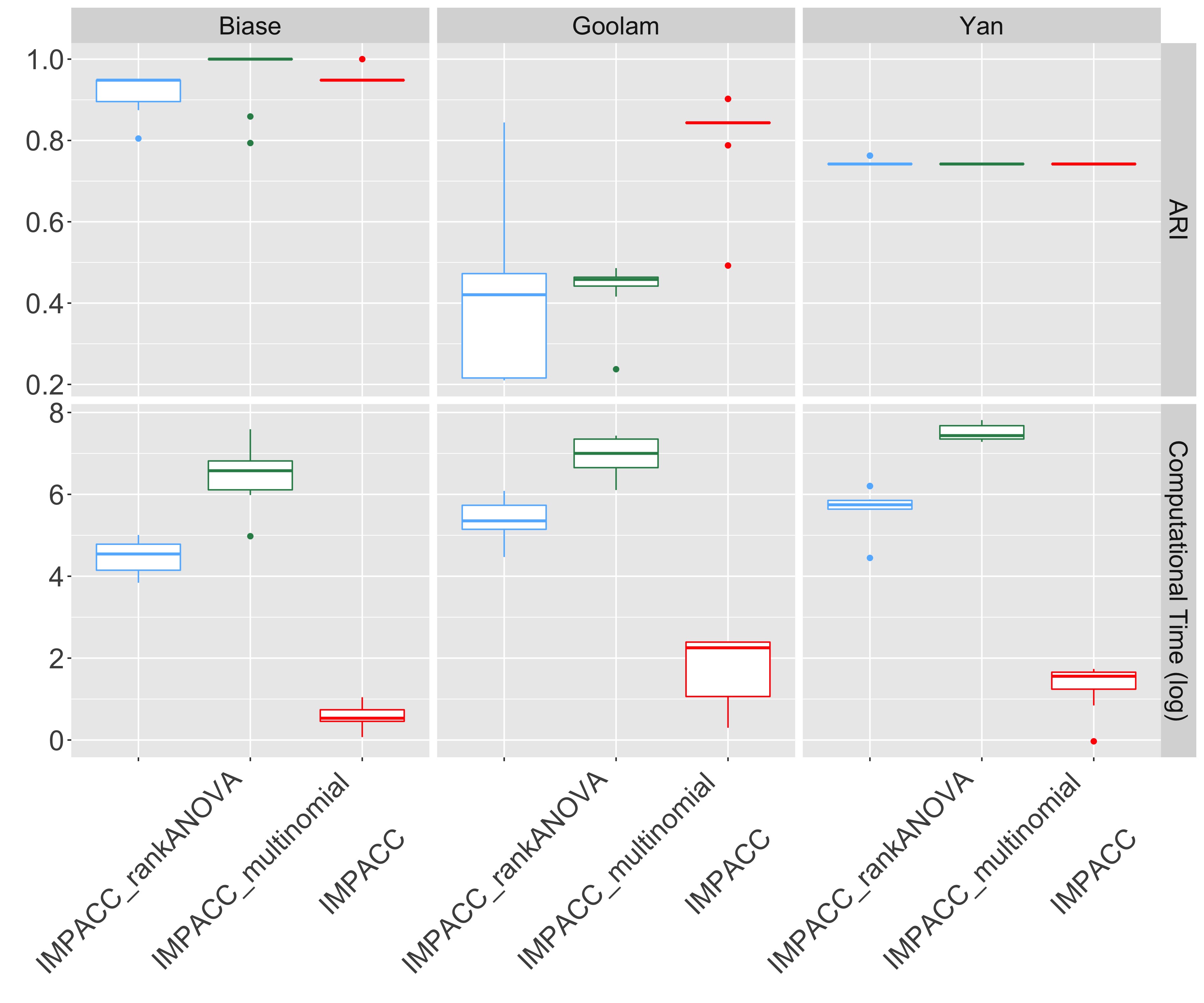


Figure 13: Compare ARI and computational time IMPACC variants, implementing simple ANOVA, rank ANOVA test or multinomial regression.


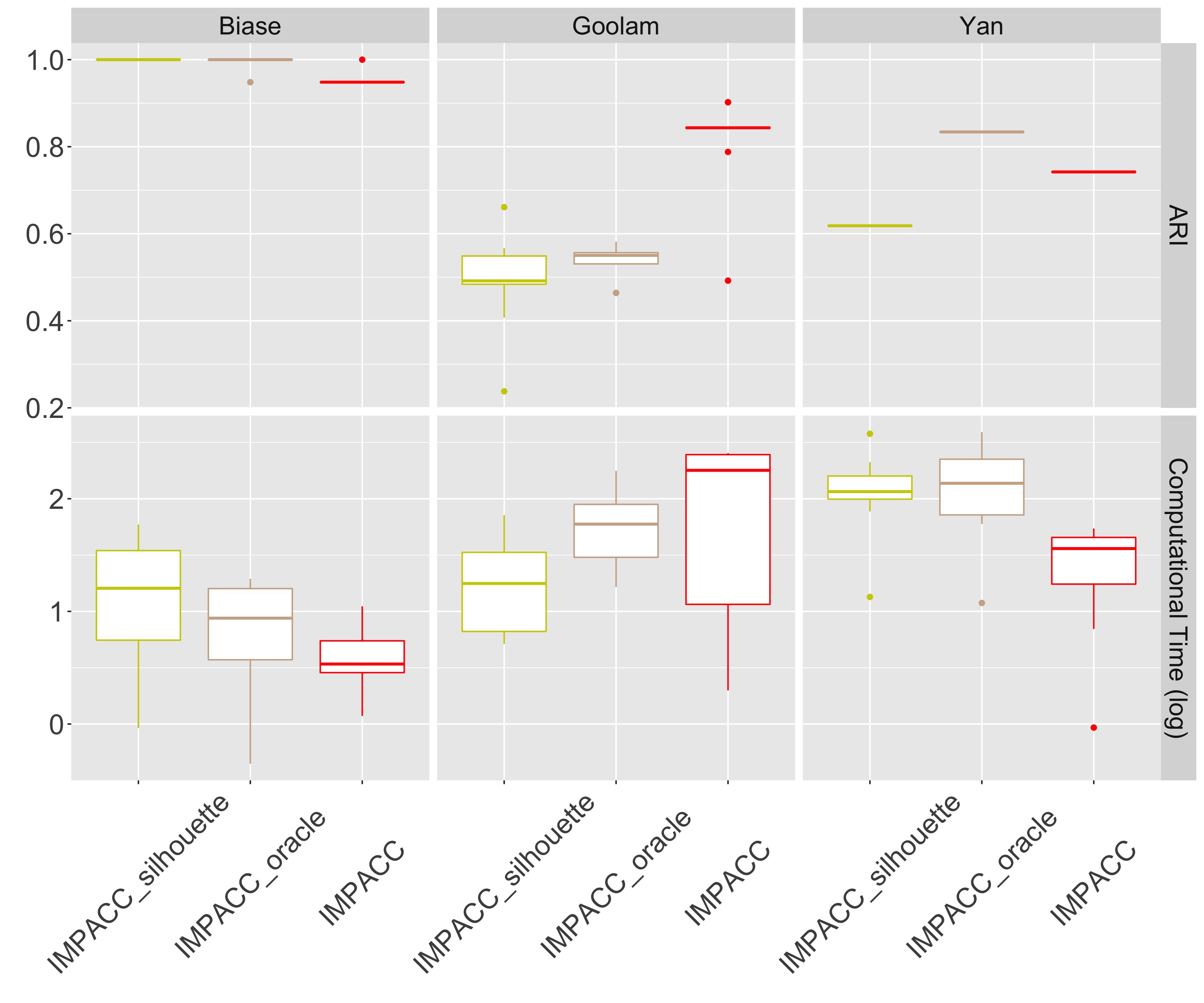


Figure 14: Compare ARI, computational time and F1 of IMPACC variants on selecting K.

# H A study on Hyper-parameters and Hyper-parameter Tuning

There are 3 hyper-parameters in MPCC and 5 additional hyper-parameters in IMPACC. However, only minipatch size-related parameters *n* and *m* can significantly influence running time and model accuracy. Therefore, choosing a pair of *n* and *m* that is relatively small while maintaining good performance would be critical. We find that our framework is fairly robust by setting *m* = 0*.*1 and *n* = 0*.*25 in empirical studies. And in the case of *N <* 100, we would set *n* = 0*.*5 to ensure enough observations in a minipatch. In addition, the remaining hyper-parameters do not need to be tuned, because the model performance is either insensitive and stable to a wide range of parameter choices, or is optimal with one universal value in all cases. Since our models are quite robust and can generate satisfying performance with default parameter settings, we can remarkably reduce computational costs. Hyper-parameters and their default values are summarized in Table 3, 4, 5, and comparisons on learning accuracy versus number of iterations for different levels of hyper-parameters on two real genomics data are specified in Figure 15-22.

Besides, We propose a data-driven way to conduct parameter tuning based on the consensus matrix. For example, in the selection of minipatch size, we found that our models can yield optimal learning performance by choosing the minimum *m* and *n* pair such that the maximum confusion value of the final consensus matrix is less than 0*.*01.

# I Clustering Results on Real Data Sets

Table 6 summarizes the mean and standard deviation of 10 realizations of clustering results on real data sets.

| Parameter | Description Default value | | | Range |
| --- | --- | --- | --- | --- |
| *h* | 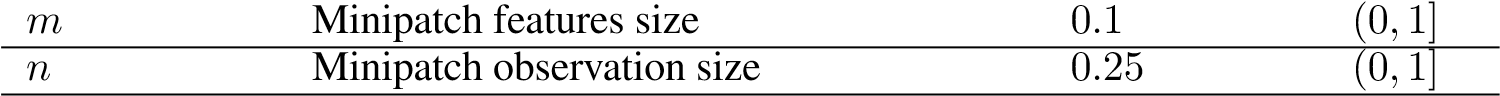  Cutoff quantile on hierarchical tree height 95% | | | (0*,*1] |
|  | Table 3: Hyper-parameters in MPCC framework ( | | Algorithm 1). |  |
| Parameter | Description | | Default value | Range |
| {*η*} | p-value percentile cutoff | | 5% | [0*,*1] |
| *α_F_* | learning rate (feature) | | 0.5 | [0*,*1] |
| {*τ*} | high importance cutoff (*mean*+*τsd*) 1 | | | [0*,*∞) |
|  | Table 4: Hyper-parameters in adapti | ve feature sampling scheme. | |  |
| Parameter | Description | Default value | | Range |
| *α_I_* | learning rate (observation) | 0.5 | | [0*,*1] |
| {*θ*} | high uncertainty cutoff | 95% weight quantile | | [0*,*1] |

Table 5: Hyper-parameters in adaptive observation sampling scheme.


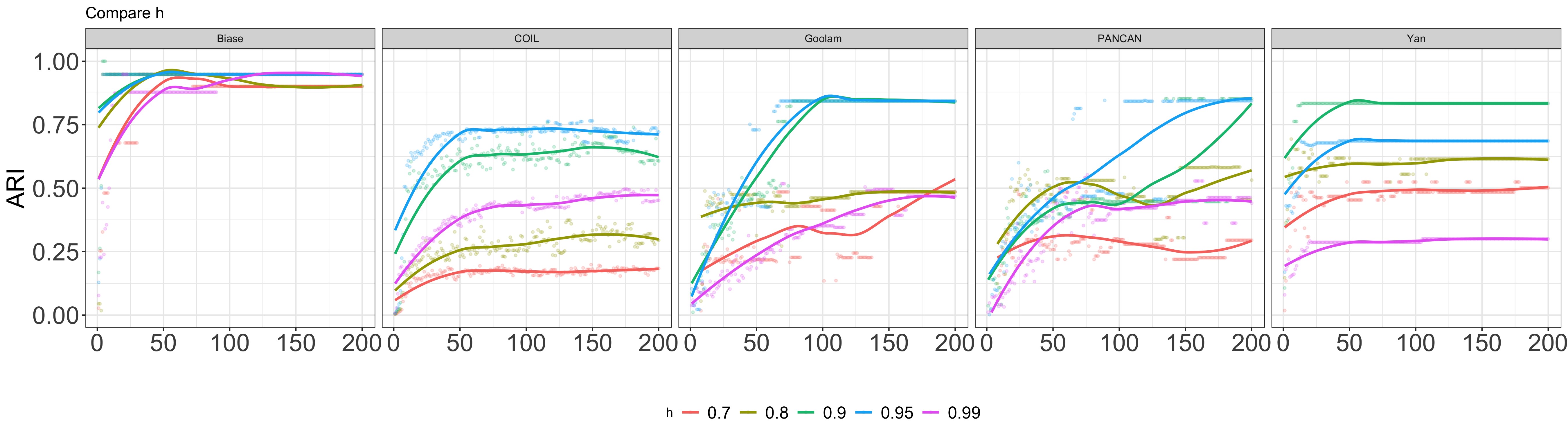


Figure 15: Clustering performance measured by ARI with different values of hierarchical tree cutoff *h* in IMPACC using brain cells and PANCAN data. Methods with *h* = 0*.*95 setting have significantly better clustering performance. So we use *h* = 0*.*95 as default value.


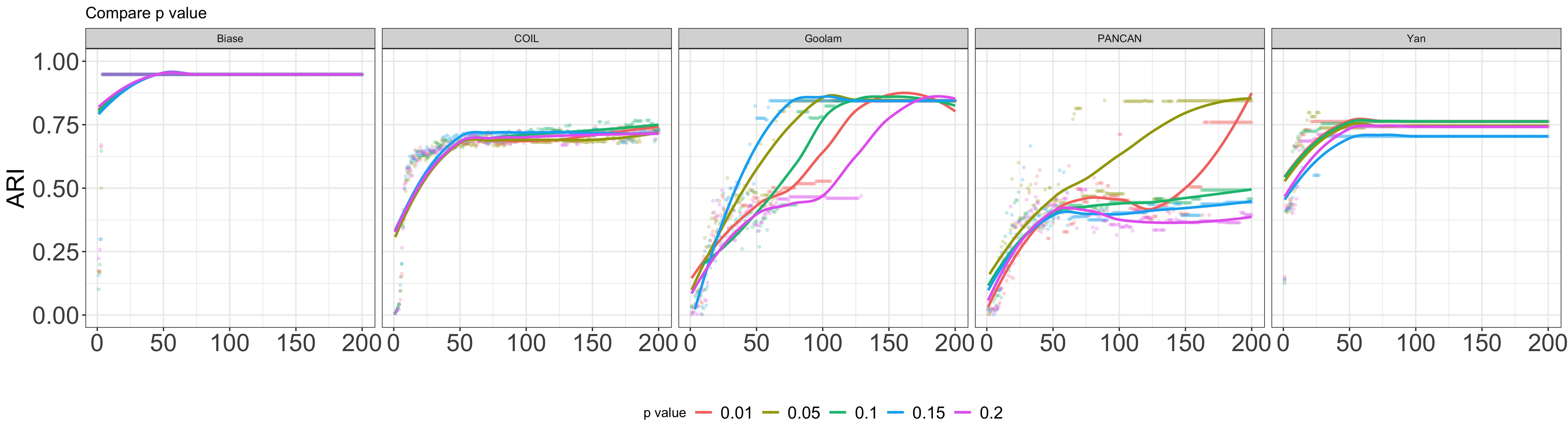


Figure 16: Clustering performance measured by ARI with different values of p-value percentile cutoff {*η*} in IMPACC using brain cells and PANCAN data. Clustering accuracy is not significantly different with various {*η*} settings.


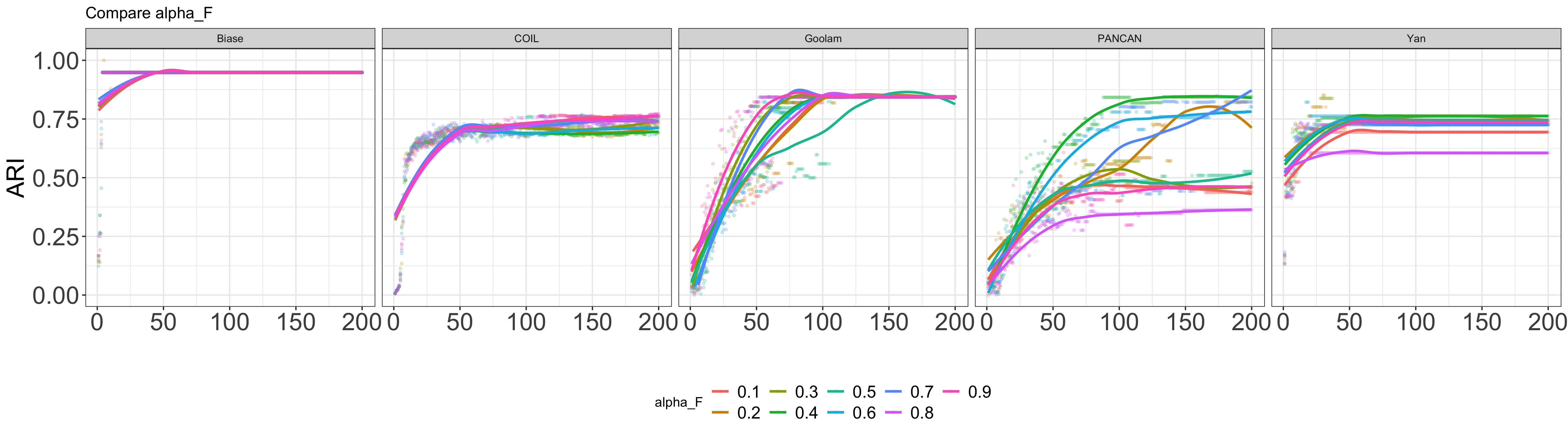


Figure 17: Clustering performance measured by ARI with different values of feature learning rate *α_F_* in IMPACC using brain cells and PANCAN data. Clustering accuracy is not significantly different with various *α_F_* settings.


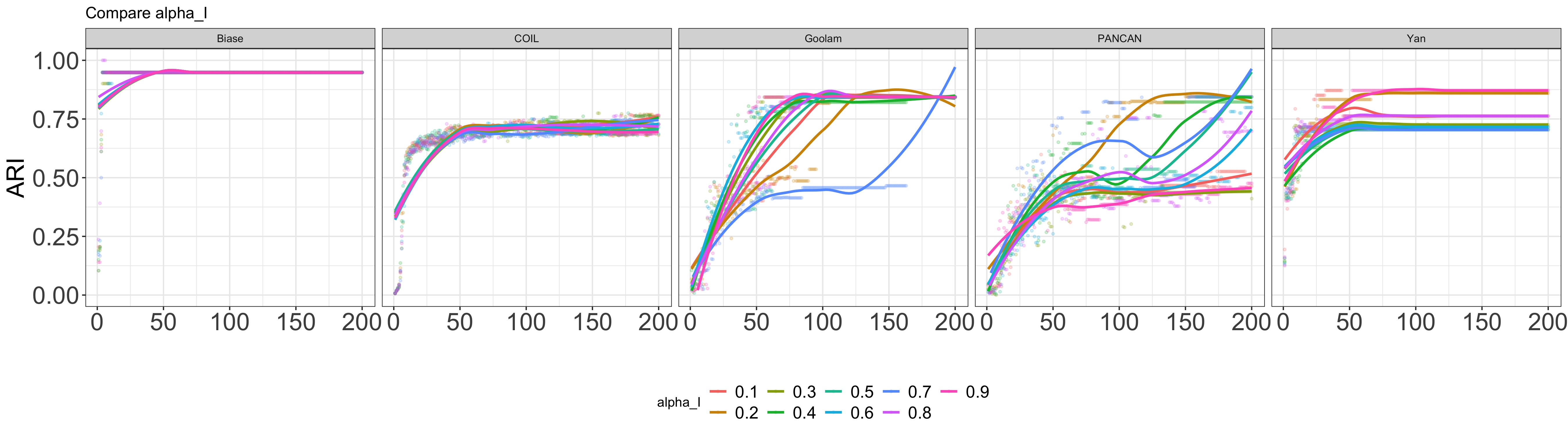


Figure 18: Clustering performance measured by ARI with different values of observation learning rate *α_I_* in IMPACC using brain cells and PANCAN data. Clustering accuracy is not significantly different with various *α_I_* settings.


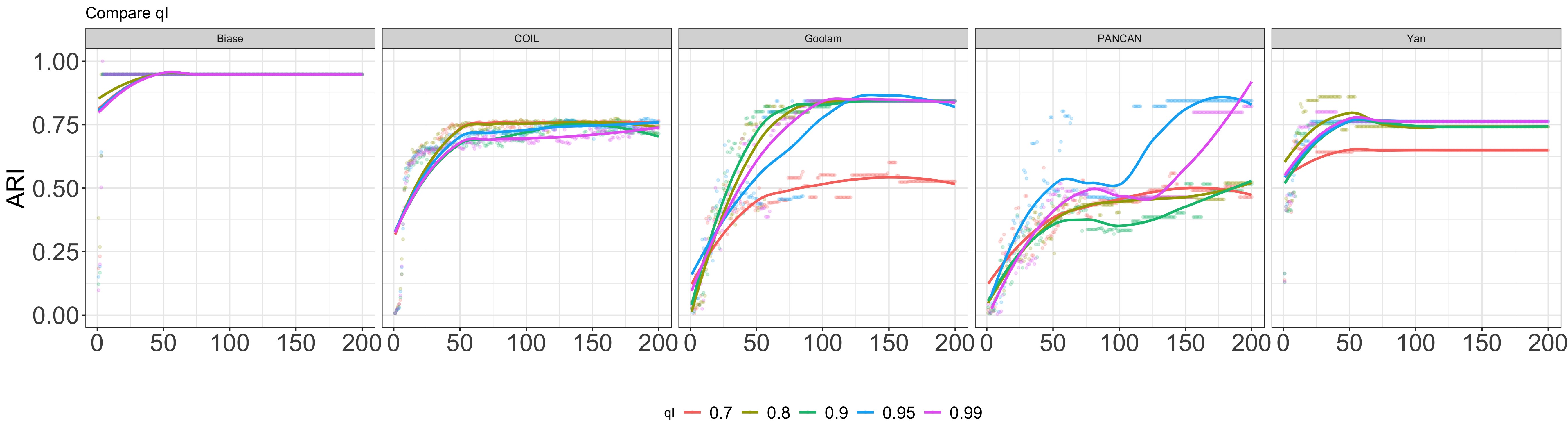


Figure 19: Clustering performance measured by ARI with different values of high uncertainty cutoff {*θ*} in IMPACC using brain cells and PANCAN data. Clustering accuracy is not significantly different with various {*θ*} settings.


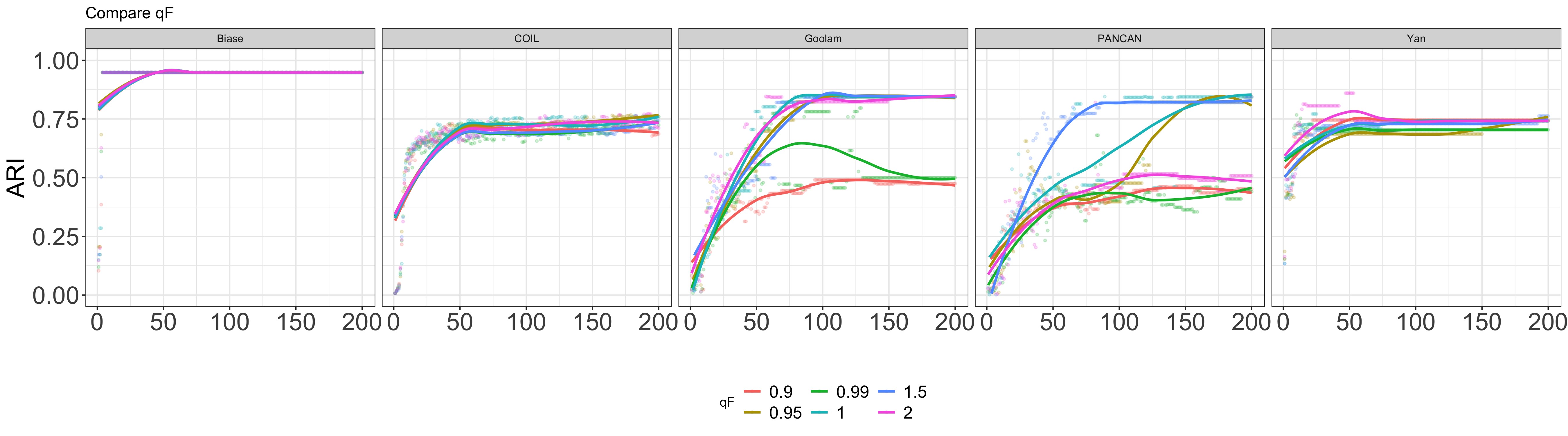


Figure 20: Clustering performance measured by ARI with different values of high importance cutoff {*τ*} in IMPACC using brain cells and PANCAN data. Clustering accuracy is not significantly different with various {*τ*} settings.


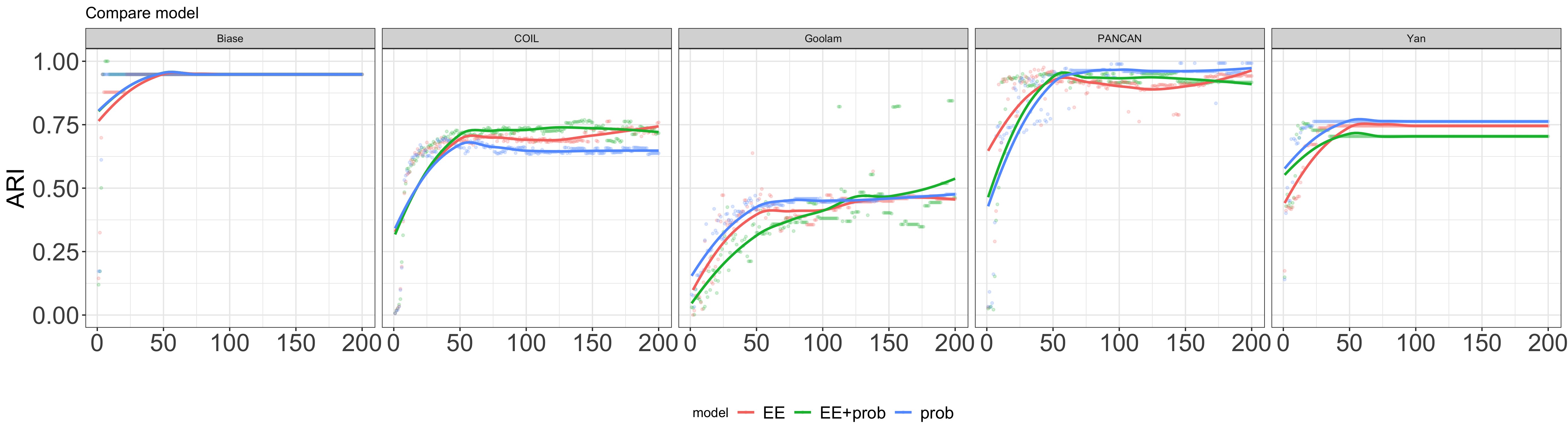


Figure 21: Clustering performance measured by ARI with different adaptive sampling schemes in IMPACC using brain cells and PANCAN data. The schemes includede are probabilistic sampling scheme (*prob*), proposed *EE* + *Prob* scheme, and *EE* scheme from [29]. Clustering accuracy is not significantly different with different adaptive sampling methods.


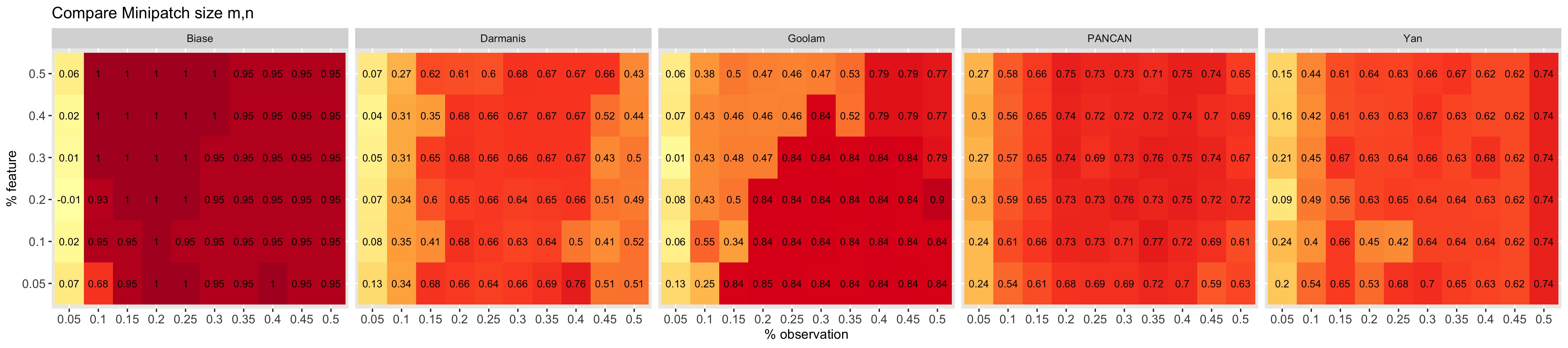


Figure 22: Clustering performance measured by ARI with different values of minipatch size *m* and *n* in IMPACC using brain cells and PANCAN data.

|  |  | | ARI |  |  |  |  | Time (s) |  |  |
| --- | --- | --- | --- | --- | --- | --- | --- | --- | --- | --- |
|  | PANCAN | Biase | Goolam | Yan | COIL20 | PANCAN | Biase | Goolam | Yan | COIL20 |
| IMPACC (HC) | 0.991 (0.001) | 0.953 (0.016) | 0.815 (0.118) | 0.742 (0) | 0.74 (0.005) | 33.379 (14.925) | 1.849 (0.556) | 7.602 (4.168) | 4.17 (1.539) | 70.297 (39.085) |
| IMPACC (Spec) | 0.99 (0.002) | 0.953 (0.016) | 0.829 (0.029) | 0.742 (0) | 0.663 (0.027) |  |  |  |  |  |
| MPCC (HC) | 0.982 (0.017) | 0.948 (0) | 0.66 (0.133) | 0.742 (0) | 0.717 (0.021) | 21.897 (6.527) | 0.093 (0.091) | 4.73 (1.372) | 2.189 (0.713) | 52.446 (35.016) |
| MPCC (Spec) | 0.991 (0.002) | 0.948 (0) | 0.682 (0.077) | 0.772 (0.064) | 0.672 (0.01) |  |  |  |  |  |
| Consensus (HC) | 0.754 (0.008) | 0.953 (0.016) | 0.452 (0.047) | 0.834 (0) | 0.673 (0.016) | 75.377 (18.527) | 0.121 (0.021) | 1.797 (0.163) | 0.967 (0.176) | 557.623 (392.506) |
| Consensus (Spec) | 0.774 (0.008) | 0.953 (0.016) | 0.684 (0.01) | 0.763 (0) | 0.67 (0.011) |  |  |  |  |  |
| sparseKM | 0.981 (0) | 1 (0) | 0.459 (0) | 0.736 (0.131) | 0.441 (0.013) | 1044.875 (143.35) | 46.636 (3.447) | 141.162 (4.594) | 68.011 (3.591) | 95.572 (23.097) |
| sparseHC |  | 0.342 (0.008) | 0.514 (0) | 0.777 (0) |  |  | 14.883 (2.936) | 86.904 (2.554) | 22.97 (1.556) |  |
| Seurat |  | 0.66 (0) | 0.447 (0) | 0.548 (0) |  |  | 0.922 (0.077) | 1.412 (0.051) | 0.791 (0.103) |  |
| SC3 |  | 0.948 (0) | 0.687 (0) | 0.731 (0) |  |  | 75.29 (3.253) | 73.234 (0.992) | 68.598 (1.853) |  |
| tSNE+KMeans | 0.983 (0) | 0.509 (0) | 0.317 (0) | 0.736 (0) | 0.619 (0) | 7.853 (0.673) | 0.288 (0.023) | 1.598 (0.033) | 0.514 (0.017) | 133.09 (18.195) |
| tSNE+HC | 0.991 (0) | 0.948 (0) | 0.3 (0) | 0.671 (0) | 0.685 (0) | 7.864 (0.674) | 0.287 (0.023) | 1.598 (0.034) | 0.514 (0.017) | 3.543 (0.676) |
| tSNE+spectral | 0.803 (0) | 0.948 (0) | 0.307 (0) | 0.666 (0) | 0.787 (0) | 12.598 (1.442) | 0.411 (0.148) | 1.685 (0.035) | 0.594 (0.095) | 3.578 (0.682) |
| tSNE+KMedoid | 0.98 (0) | 0.948 (0) | 0.354 (0) | 0.641 (0) | 0.727 (0) | 8.008 (0.683) | 0.287 (0.023) | 1.6 (0.034) | 0.516 (0.018) | 20.255 (6.219) |
| KMeans | 0.795 (0) | 0.948 (0) | 0.493 (0) | 0.544 (0) | 0.771 (0) | 2.67 (0.218) | 0.045 (0.007) | 0.326 (0.013) | 0.107 (0.011) | 7.04 (1.654) |
| HClust | 0.756 (0) | 0.948 (0) | 0.433 (0) | 0.763 (0) | 0.54 (0) | 57.236 (9.584) | 0.057 (0.002) | 1.116 (0.076) | 0.201 (0.011) | 0.201 (0.04) |
| Spectral | 0.734 (0) | 0.948 (0) | 0.381 (0) | 0.473 (0) | 0.65 (0) | 4.817 (0.508) | 0.118 (0.063) | 0.393 (0.015) | 0.181 (0.044) | 2.097 (0.718) |
| KMedoid | 0.761 (0) | 1 (0) | 0.676 (0) | 0.743 (0) | 0.447 (0) | 58.955 (4.48) | 0.066 (0.003) | 1.212 (0.054) | 0.214 (0.026) | 12.491 (3.95) |

Table 6: Clustering performance (ARI) and computation time in seconds on real data sets with known cluster labels, followed by standard deviation of 10 realizations.

# References

1. Luke Zappia, Belinda Phipson, and Alicia Oshlack. Splatter: simulation of single-cell rna sequencing data. *Genome biology*, 18(1):1–15, 2017.
2. Mubeen Goolam, Antonio Scialdone, Sarah JL Graham, Iain C Macaulay, Agnieszka Jedrusik, Anna Hupalowska, Thierry Voet, John C Marioni, and Magdalena Zernicka-Goetz. Heterogeneity in oct4 and sox2 targets biases cell fate in 4-cell mouse embryos. *Cell*, 165(1):61–74, 2016.
3. Liying Yan, Mingyu Yang, Hongshan Guo, Lu Yang, Jun Wu, Rong Li, Ping Liu, Ying Lian, Xiaoying Zheng, Jie Yan, et al. Single-cell rna-seq profiling of human preimplantation embryos and embryonic stem cells. *Nature structural & molecular biology*, 20(9):1131–1139, 2013.
