# Supplementary material for: Fast and interpretable consensus clustering via minipatch learning
Source: PLoS Comput Biol. 2022 Oct 3;18(10):e1010577. doi: 10.1371/journal.pcbi.1010577 (PMC9560608; doi:10.1371/journal.pcbi.1010577)
Supplement: Attachment — Submitted filename: Review comments.pdf [file pcbi.1010577.s004.pdf]

In this manuscript, the authors adapt their previously introduced minipatch learning approach to be used for unsupervised learning, within a consensus clustering framework, through a new algorithm entitled IMPACC (Interpretable MiniPatch Adaptive Consensus Clustering). As the authors argue, computational efficiency is a key advantage of using minipatches (i.e., small slices of the data matrix comprising subsets of both observation and features) to ensemble cluster co-occurrences from multiple clustering runs. This is particularly relevant for large scale biological datasets such as single cell RNA-seq data. Methodologically, IMPACC introduces adaptive strategies for sampling minipatches in each ensemble iteration, leveraging information from prior iterations to overweight the inclusion of observations and features that are informative for the "true" underlying clusters. The authors then use both synthetic and real datasets to evaluate the performance of their algorithm against other established methods and to argue that IMPACC yields more accurate and interpretable cluster solutions.

The adaptive sampling strategies proposed are reasonable. Innovative elements include the specific method for constructing and updating the observation and feature weights used for resampling, as well as the mixing of weighted and unweighted sampling to minimize sampling bias (some limitations of using ANOVA in feature sampling are discussed in the Specific Comments below). My biggest concern about the manuscript is the performance evaluation framework. For that purpose, the authors utilize both synthetic and real datasets. The generative model for the synthetic data is rather narrow. It uses fixed values for the number and size of clusters and makes overly constraining assumptions about the structure of the correlation between features. Without results for a reasonably wide range of parameter settings, it is difficult to generalize conclusions about the algorithm's performance. The issue of the feature correlation structure is particularly vexing. Since scRNA-seq is a key target application, the authors should consider using real data to inform assumptions about feature correlation. Real datasets are in fact used for evaluating the ability of IMPACC to recapitulate "ground-truth" clusters. However, as far as I can tell (the authors provide near-zero description of the dataset details beyond a summary table), the ground truth is itself just the result of the computational clustering analysis performed by the original studies. This may not be the best approach to assess performance since, to quote the authors, "for huge and sparse data sets common with genomic sequencing technologies, clustering methods can suffer from unreliable results". In other words, they could potentially be evaluating IMPACC on its ability to recapitulate incorrect answers. Compounding the issue, these datasets are used in the appendix for deriving default values for key IMPACC parameters, such as the observation and feature sample sizes used during the consensus clustering iterations.

There is also lack of adequate detail to properly assess some of the conclusions drawn. E.g., in Table 3, KEGG pathway enrichment analysis results are presented demonstrating that pathways and enrichment scores acquired by using the top gene features identified by IMPACC are more informative than what is gotten when using the (significantly fewer) top genes from sparseKM and sparseHC. But this may just be a consequence of how the other tools were run. E.g., using a small lasso penalty for sparseKM will constrain the algorithm to select a small set of features; this set can still lead to accurate clustering but will be devoid of highly correlated genes, i.e., precisely the genes that would be beneficial in enrichment analysis. This is not necessarily a limitation. In practice, the typical approach is to follow clustering with differential gene expression analysis, to identify genes that are differentially expressed in a given cluster, and then use those genes for pathway analysis.

Finally, attention needs to be paid to the overall quality of the manuscript. Variables in pseudocodes are used without definition. In most cases, the definition is hidden in the appendix. But there are also variables that are just never defined (e.g., "E" in Algorithm 3). In some places indices appear to be missing (e.g., "u" in the nominator of the expression in step 2, of Algorithm 2). There are occasional discrepancies between the text and the pseudo code. E.g., the text states "$\gamma(t) \in [0.5, 1]$ is a monotonically increasing parameter that controls sampling size". But the value $\gamma(t)$ appears constant in the code for Algorithm 3. And almost uniformly, initialization values for variables are not provided. And as mentioned above, there is lack of detail about precisely how the comparisons to competing clustering algorithms were performed.

In summary, while the methodology presented can conceivably offer the benefits claimed, there is not enough evidence to properly assess some of the performance claims.

Specific Comments

- The rationale behind the specific parameter choices for the generation of the synthetic data should be presented. Otherwise they seem arbitrary.
- Data presented in Figure 15 of the Appendix, raise some questions. Specifically, for a given selection of n (size of feature sample), one would expect that clustering performance would monotonically improve with an increase in m (size of observation sample). This is clearly not the case for the PANCAN dataset, where, e.g., for n =-0.3, there is a precipitous drop in ARI from almost 1 (m = 0.2) to 0.75 (m = 0.5). Why is this?
- Feature weighting in Algorithm 4 uses simple ANOVA, without considering feature correlation/collinearity. Given the highly correlated nature of gene expression, this approach could up-weight highly correlated features at the expense of other possibly informative features. The synthetic dataset, as constructed, would not be able to surface this limitation due to its artificially over-constrained feature correlation structure.
- For the comparisons against the 3rd party clustering algorithms, details of precisely how these algorithms were executed need to provided (e.g., in the appendix). Otherwise it is not possible to assess if these analyses were performed properly. For instance, for the neoplastic dataset, the authors report a rather dismal ARI of 0.346 for the very clustering method (tSNE + kMeans) used in the publication introducing and analyzing this dataset (PMID: 29091775) and which, presumably, generated the cluster labels. This is highly surprising .
- The real data sets used should be described in more detail. The authors should also clarify the statement "We apply our methods to three RNA-seq data sets and one image data set with known cluster labels", and specifically what is the source of the "known cluster labels". The details in Table 1 should also be double checked for accuracy. E.g., the GEO accession number provided for the PANCAN data set is likely wrong. Similarly, for the other GEO datasets, the number of observations listed in Table 1 is different from the number of samples listed in GEO for the indicated GSE ids. The source of the discrepancy should be explained.
- The clustering problem statements reads: "We assume that the observations can be separated into K non-overlapping and exhaustive clusters; our goal is to find these clusters." This is too informal. Any set of >= K observations can be separated arbitrarily into K non-overlapping and exhaustive clusters. More precise language should be used, e.g., recasting the problem in optimization terms.
