# Supplementary material for: Fast and interpretable consensus clustering via minipatch learning
Source: PLoS Comput Biol. 2022 Oct 3;18(10):e1010577. doi: 10.1371/journal.pcbi.1010577 (PMC9560608; doi:10.1371/journal.pcbi.1010577)
Supplement: Attachment — Submitted filename: response.pdf [file pcbi.1010577.s005.pdf]

Response to reviews for PCOMPBIOL-D-21-02086: Fast and Interpretable Consensus Clustering via Minipatch Learning

May 10, 2022

1 Response to Reviewer and AE Comments

We thank the reviewers and the action editor for their careful reading and thoughtful feedback. We have made several changes to the paper in response to these comments and believe the paper is significantly stronger. We summarize our major changes below and then provide our detailed response to the reviewers' comments in the following sections. The main changes in the manuscript are all marked in blue for clarity. In summary, we made the following major changes in our empirical study:

New synthetic data simulation with autoregressive covariance. We designed a new simulation study with a $AR(1)$ autoregressive covariance structure, which has greater feature correlations and is a better representative of high dimensional bioinformatics data.

Evaluation on Feature Importance in Synthetic Data. We present two approaches to compare the feature selection accuracy among IMPACC, sparseKM, and sparseHC in the study of synthetic data. IMPACC provides feature importance scores ranging in $[0, 1]$, and sparseKM and sparseHC generate sparse feature weights with zero values for unimportant features. The oracle method selects the top 25 features (the oracle number of signal features) with the highest importance score in IMPACC or the highest non-zero weights in sparseKM and sparseHC. And the data-driven feature selection is to select features with importance scores are higher than the mean plus one standard deviation of all scores in IMPACC, and choose all the features with non-zero weights in sparseKM and sparseHC [Witten and Tibshirani, 2010].

New gold standard single-cell RNA-seq data with known cell labels. We replace our previous two single-cell data sets with three new benchmark single-cell data sets: Biase [Biase et al., 2014], Goolam[Goolam et al., 2016], and Yan[Yan et al., 2013]. The cluster information of the three single-cell RNA-seq data is known because these data sets are generated from cells of various development stages. The Biase data is generated from 49 single cells composed of 1-cell (zygote), mid-stage 2-cell, and 4-cell mouse embryos. The Goolam data investigates gene expression patterns in the pre-implantation development of mouse embryos, including cells isolated from the 2-cell stage to the 32-cell stage. And the Yan data measures gene expression of cells from human pre-implantation embryos and human embryonic stem cells at different passages. Therefore, we can use them to correctly compare IMPACC and its competitors in terms of clustering performance and enrichment analysis on selected important features.

Revised GO enrichment analysis on important features. We focus on analyzing the important features selected by IMPACC in the Yan[Yan et al., 2013] data set. We show that IMPACC is able to provide accurate and reliable interpretations of scientifically important genes as well as biologically meaningful GO enrichment analysis; these results match the original paper's scientific conclusions in which the cell types are known.

Appendix

1. We add another synthetic data generated from a new real single-cell RNA-seq data set using the *splatter* single-cell simulation method [Zappia et al., 2017];

2. We add comparisons on different hyper-parameter values on all real data sets;

3. We test the performance of IMPACC using different alternatives of feature importance measurement: 1) ANOVA on rank, 2) multinomial regression. And approaches to determine the number of clusters in a minipatch: 1) select cluster number with the highest silhouette score, 2) oracle K.

4. We put the complete lists of GO-enriched terms identified by IMPACC, SC3, and sparseKM in the Appendix, along with the GO enrichment analysis reported in Yan et al. [2013] for EPI-specific genes compared to all the other cells after the 8-cell stage.

2 Reply to reviewer 1

1. **Throughout the manuscript, authors claim that in high-dimensional biological data, we expect only a small subset of features to be relevant for determining clusters. Although this can be true in some cases, I can think of many scenarios where this is not true (even in some of the datasets they analyze). For example, in a biopsy of a tumor within a normal tissue, the tumor cells have a vastly different gene expression profile. Even within a normal tissue, let's say bone marrow, we expect to find a wide variety of cell types, each one with many distinct genes expressed and common ones expressed at different levels. This is not to say that their method is not applicable to such datasets but I would expect that the authors consider and discuss the implications in such cases.**

   We thank the reviewer for the question. IMPACC can achieve better performance when the data set is more sparse. As the reviewer discussed, it is still applicable to the scenario when the sparsity level is low. However, IMPACC would show less advantage in selecting differential expressed genes in such cases. We would recommend using MPCC (MiniPatch with Consensus Clustering) with random minipatch sampling, which has comparable clustering accuracy but faster computational speed than regular consensus clustering, as shown in the *D.3 No Sparse Block-Diagonal Simulation* section in Appendix.

   2. In at least two instances (lines 141 and 248), the authors choose a relatively simple methodology so that the overall algorithm is fast enough. To test that this is the best choice (not in terms of speed but in terms of accuracy), I would expect to see a side-by-side comparison with additional tests/methods and show that speed is not compromising accuracy.

   We thank the reviewer for this suggestion. We constructed variants of IMPACC by replacing simple methodologies with more complex ones. Specifically, we focus on the following two aspects.

   (a) How to evaluate association between features and cluster labels in a minipatch. Our current IMPACC evaluates the level of association between each feature and the estimated cluster labels on that minipatch by a one-way ANOVA test. We first consider a more complex alternative: a non-parametric ANOVA (Kruskal-Wallis test), which relaxes normality assumptions. Secondly, we agree with reviewer 2 that the ANOVA test does not consider feature collinearity. Therefore, we propose to conduct a multinomial regression of features to predict cluster assignments, which can account for feature correlations. Specifically, one feature's association level is calculated as the mean of predicted probabilities on the cluster assignments from the

fitted multinomial regression.

(b) How to determine the number of clusters k in a minipatch. Our current IMPACC method cuts the hierarchical tree at the h quantile (typically set to be 0.95). We consider two alternatives: 1). for a given range of k values, we select the one that can generate the highest silhouette score; 2). use the oracle number of clusters K as in regular consensus clustering and SC3.

With other parts of the algorithm being the same, we apply the proposed variants to the three benchmark single-cell RNA-seq data. As shown in Figure 1, IMPACC variants that implement rank ANOVA test or multinomial regression do not demonstrate advantages in clustering accuracy, measured by ARI. Also, the variants have much more significant computational burdens than the IMPACC with a simple ANOVA test.

In addition, Figure 1 compares different alternatives to determine the number of clusters in a minipatch. The IMPACC variants can have slightly higher ARI in some cases, but can not outperform IMPACC, which cut the hierarchical tree at a default 0.95 quantile, in clustering accuracy and computational time simultaneously. Therefore, we can conclude that even though IMPACC implements simple procedures in the algorithm, it does compromise accuracy for computational efficiency. Some may wonder why the variant with oracle K has greater computational time in some of the data sets. It is because we implement early stopping criteria based on the changes in the consensus matrix, and the variant with oracle K would take more iterations to reach a stable consensus matrix.

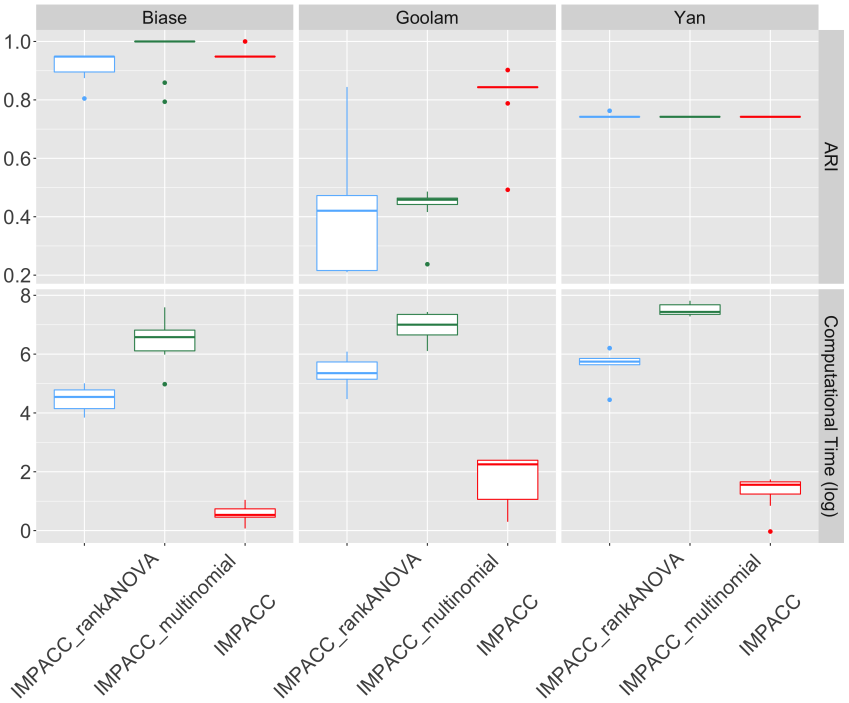

Figure 1: Compare ARI and computational time IMPACC variants, implementing simple ANOVA, rank ANOVA test or multinomial regression

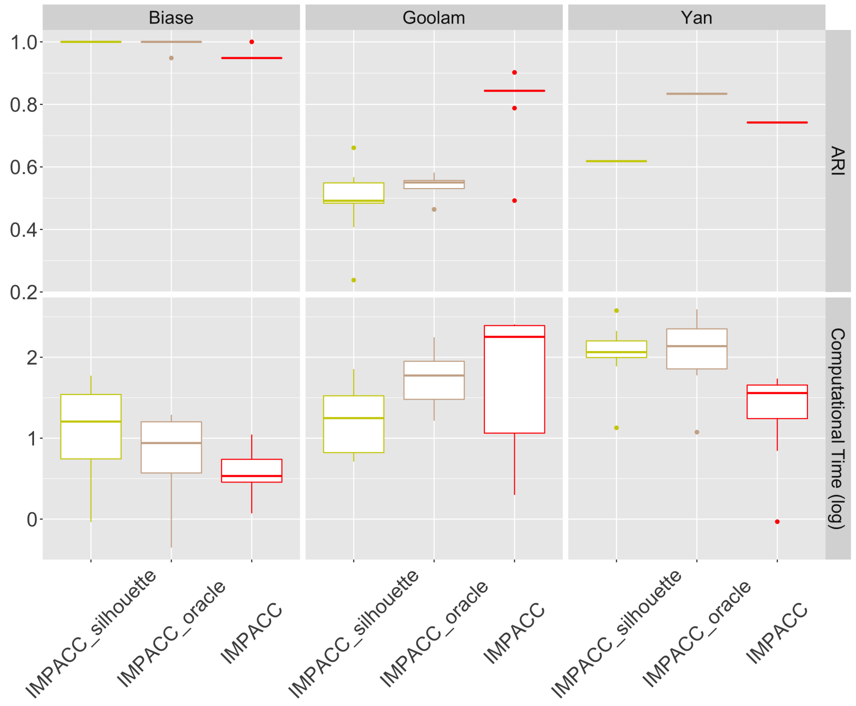

Figure 2: Compare ARI, computational time and F1 of IMPACC variants on selecting K.

3. **Lines 131-151 deal with the problem of accuracy. I am not sure that I understand how this approach specifically addresses the problem of accuracy. By the end of this paragraph, I can understand the methodology but not how it achieves better accuracy (only how it proposes to do so).**

We thank the reviewer for the question. Line 131-151 discuss how we determine the number of clusters in a minipatch. The regular consensus and SC3 use the given fixed K for each iteration. However, since a minipatch is built by subsampling a small set of observations and features, the number of clusters in a minipatch may vary and be different from the oracle K, especially in the case of unbalanced clusters. By cutting the fitted hierarchical tree of the minipatch, the number of clusters is adaptive to the clusters present in that minipacth, and it would be more accurate than using a fixed number of clusters as in regular consensus clustering and SC3. As shown in response to the second point of Reviewer 1, we explored alternative ways to determine the number of clusters in a minipatch. Figure 1 compares the clustering accuracy in the three benchmark single-cell RNA-seq data set, and our proposed IMPACC can generate

relatively high and stable clustering accuracy.

4. **The biological interpretations are extremely weak (lines 342-355). Authors perform KEGG pathway analysis (not described with which tool, statistical thresholds?) and argue that some pathways are important in one setting vs. the other. This is a subjective analysis and I can argue for the opposite in some cases: Why is insulin secretion important in IMAPCC of Table 3 when we are focusing on brain cells? Why is this not a false positive? Authors need to look at the genes within each pathway in more detail. They also need to justify their findings biologically and compare them to the ones reported in the original papers that generated the data they use. In addition, they need to perform some basic correlation analysis of importance weights, pathway fold enrichments (not just p values) among the three tested approaches.**

We thank the reviewer for this suggestion. We revised the biological interpretation part and conducted a more solid analysis of the top features selected by IMPACC in the Yan[Yan et al., 2013] data set. Firstly, we show that the important genes selected by IMPACC are actually differentially expressed genes, as shown in Figure 1. Since the feature support set in IMPACC is constructed by including features that demonstrate different expressions across clusters, we can identify differentially expressed genes from the final feature importance scores. We also propose a data-driven way to set the cutoff as the mean plus one standard deviation of all scores. Taking the Yan [Yan et al., 2013] data set as an example, IMPACC selects 466 differentially expressed genes using the data-driven cutoff, and the full list of genes can be found in Table S2. We plot the gene expression matrix of the top 50 differentially expressed genes determined by IMAPCC, with the subgroups defined by the final consensus matrix of IMPACC, using the oracle number of clusters $K = 7$ (separated by white vertical lines). The important genes selected by IMPACC have significantly different expressions among different clusters of cells, especially in the Morulae cluster.

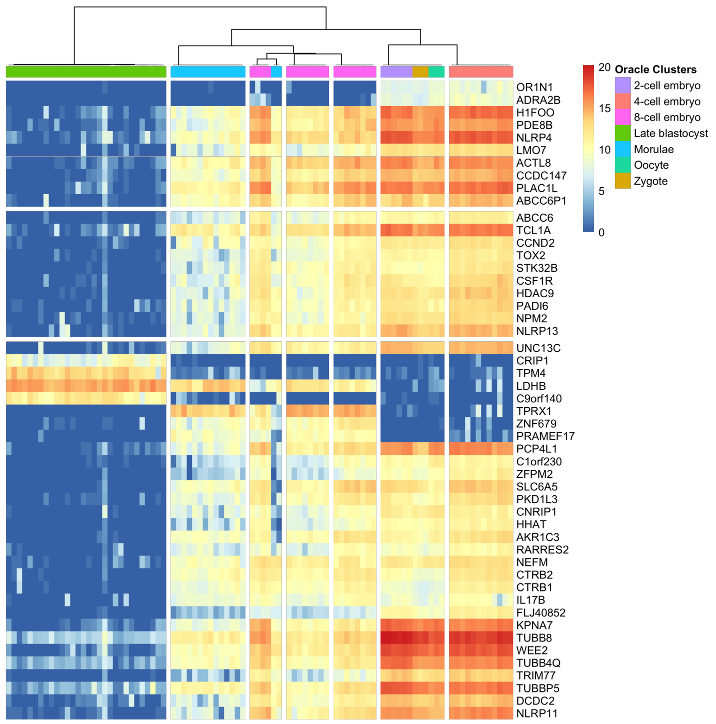

Figure 3: Gene expression matrix of the top 50 differentially expressed genes in Yan data set, with subgroups defined by the final consensus matrix of IMPACC.

Then we conducted GO enrichment analysis on the selected differentially expressed genes, and confirmed that IMPACC is able to provide accurate and reliable interpretations of scientifically important genes as well as biologically meaningful GO enrichment analysis; these results match the original paper's scientific conclusions in which the cell types are known. The top 10 pathways with fold enrichment, p-values, and counts are illustrated in Figure 2. The enriched GO terms reported in Yan et al. [2013], the complete list of GO terms, and pathway analysis using differentially expressed genes identified by SC3 and sparseKM can be found in Appendix E and Table S3.

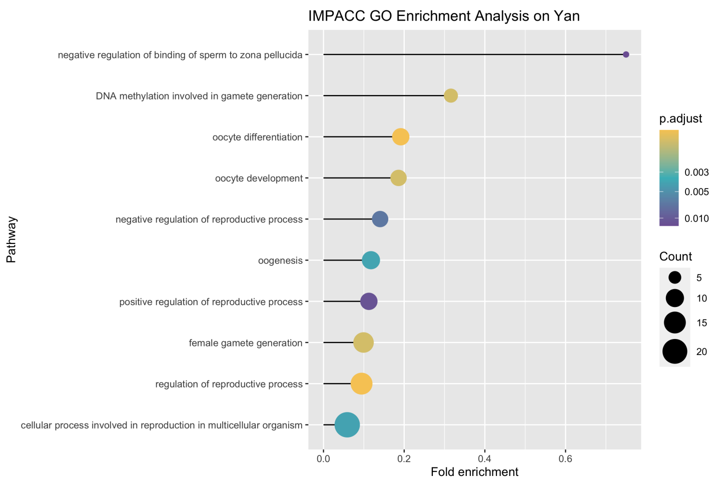

Figure 4: Top 10 pathway of GO enrichment analysis using the differentially expressed genes identified by IMPACC in Yan data set, with information on adjusted p-values, fold enrichment and count.

3 Reply to reviewer 2

1. The generative model for the synthetic data is rather narrow. It uses fixed values for the number and size of clusters and makes overly constraining assumptions about the structure of the correlation between features. Without results for a reasonably wide range of parameter settings, it is difficult to generalize conclusions about the algorithm's performance. The rationale behind the specific parameter choices for the generation of the synthetic data should be presented. Otherwise they seem arbitrary. The issue of the feature correlation structure is particularly vexing. Since scRNA-seq is a key target application, the authors should consider using real data to inform assumptions about feature correlation.

We thank the reviewer for this questions. In our synthetic data with block-diagonal covariance structure, the reason why we set the number of clusters to be 4 is that we want to generate the mean of signal features in each cluster to be $\mu_1 = \frac{SNR}{5} \cdot \mathbf{1}_{25}$, $\mu_2 = (\frac{SNR}{5} \cdot \mathbf{1}_{13}^T, -\frac{SNR}{5} \cdot \mathbf{1}_{12}^T)^T$, $\mu_3 = (-\frac{SNR}{5} \cdot \mathbf{1}_{13}^T, \frac{SNR}{5} \cdot \mathbf{1}_{12}^T)^T$, $\mu_4 = -\frac{SNR}{5} \cdot \mathbf{1}_{25}$. And we set the cluster sizes to be 4%, 16%, 24% and 56% to ensure highly unbalanced data sets, so as to better reflect the structure of real bioinformatics data set.

We agree with the reviewer that we should include a study on synthetic data with a more complex correlation structure among features. We designed two new simulation studies, and moved the simulation with block-diagonal covariance structure to the Appendix.

1. We add a sparse autoregressive simulation study, where each data set is created from a mixture of Gaussian with $AR(1)$ covariance structure. Specifically, the covariance between feature j and j' can be written as $\sigma_{j,j'} = \rho^{|j-j'|}$. This construction creates greater feature correlations and better represents high-dimensional bioinformatics data. We still have the same settings on the parameters in terms of the number of observations, features and clusters, and cluster sizes, as in the block-diagonal simulation.

   The averaged results over 10 repetitions are shown in Figure 5. IMPACC still yields the best clustering performance over all competing methods with the highest ARI in most of the SNR settings. IMPACC still achieves major advantages compared to sparse feature selection clustering strategies when comparing feature selection performance and computational efficiency. Note that the oracle and data-driven F1 scores are the same for sparseKM and sparseHC because these two methods under-select important features.

2. Another new construction of synthetic data is the *Splatter* simulation based on *Goolam* data set. Splatter[Zappia et al., 2017] single-cell simulation generates synthetic data based on a gamma-Poisson distribution, with parameters estimated from a real single-cell data set. Here we generate parameters from the *Goolam* single-cell RNA-seq data [Goolam et al.,

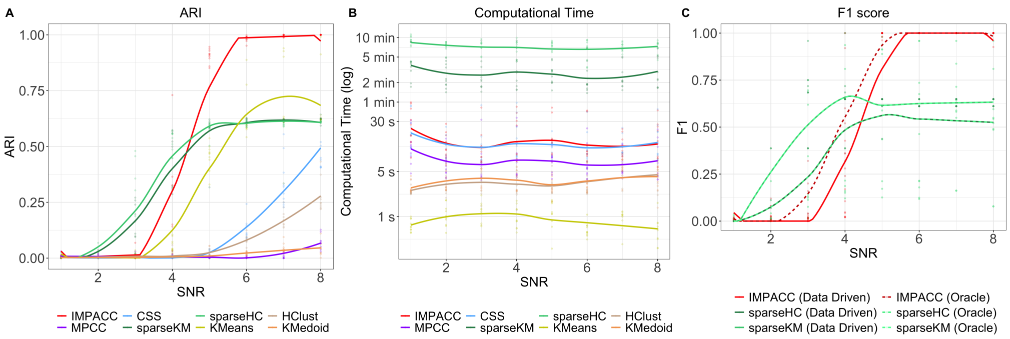

Figure 5: Clustering performance (ARI), feature selection accuracy (F1 score), and computation time on sparse synthetic data sets. (A) ARI (higher is better) of estimated grouping; (B) computation time in log seconds; (C) F1 score for signal feature estimates with oracle and data driven selection. IMPACC has superior performance over competing methods in clustering and feature selection accuracy with significant computational savings.

2016], and we set the number of observations, features and clusters to be $N = 500$, $M = 5,000$, $K = 3$, respectively. We design unbalanced cluster sizes with the percentage of observations in each cluster to be 15%, 25%, 60%, and the probability that a gene will be selected to be differentially expressed as 0.03. In addition, the difficulty of clustering can be controlled by the differential expression factors, which are produced from a log-normal distribution. Here we set the scale parameter to be 0.3 and the location parameter to be $SNR/10$, with SNR ranging from 1 to 8.

We also compare the SC3 method, which can conduct feature selection with the adjusted p-value provided for differential genes. We can select the top oracle number of features with the smallest adjusted p-values, or all features with p-values smaller than 0.05 in a data-driven way. Note that the splatter-simulated data set is a difficult task for analyzing differentially expressed genes, so the F1 score for all the feature selection methods is quite low, and IMPACC can have relatively better performance than other competitors. IMPACC still has superior performance in clustering accuracy and less computational burden than other sparse methods, as shown in Figure 2. Due to limited space in the manuscript, we put this study in Appendix.

2. Real datasets are in fact used for evaluating the ability of IMPACC to recapitulate "ground-truth" clusters. However, as far as I can tell (the authors provide a near-zero description of the dataset details beyond a summary table), the ground truth is itself just the result of the computational clustering analysis performed by the original studies. This may not be the best approach to assess performance since, to quote the authors, "for huge and sparse data sets common

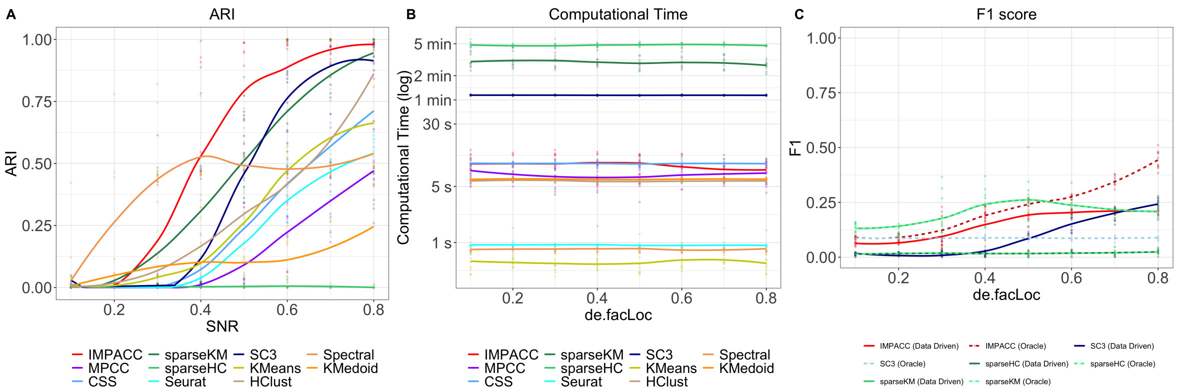

Figure 6: Clustering performance (ARI), feature selection accuracy (F1 score), and computation time on splatter synthetic data sets. (A) ARI (higher is better) of estimated grouping; (B) computation time in log seconds; (C) F1 score for signal feature estimates. IMPACC still has superior performance over competing methods in clustering accuracy with significant computational savings. Even though the feature selection accuracy is low for all the methods, IMPACC can have relatively better performance than SC3 and sparseHC

with genomic sequencing technologies, clustering methods can suffer from unreliable results". In other words, they could potentially be evaluating IMPACC on its ability to recapitulate incorrect answers

We thank the reviewer for this suggestion, and we agree with the reviewer that we might just recapitulate incorrect clustering labels in some of the real data studies. Therefore, we removed the study on our current single-cell data sets and added three new gold standard single-cell data sets: Biase[Biase et al., 2014], Goolam[Goolam et al., 2016], and Yan [Yan et al., 2013], which are also studied in the SC3 paper [Kiselev et al., 2017]. The cluster information of the three single-cell RNA-seq data are known because these data sets are generated from cells of various development stages. The Biase[Biase et al., 2014] data is generated from 49 single cells composed of 1-cell (zygote), mid-stage 2-cell, and 4-cell mouse embryos. The Goolam[Goolam et al., 2016] data investigates gene expression patterns in the pre-implantation development of mouse embryos, including cells isolated from the 2-cell stage to 32-cell stage. And the Yan[Yan et al., 2013] data measures gene expression of cells from human preimplantation embryos and human embryonic stem cells at different passages. Details are listed in Table 1. We summarize the mean of 10 realizations of clustering results on real data sets. We show that IMPACC is either the best or among the top-performing methods in each data set at discovering known clusters with the high ARI score. And IMPACC demonstrates major computational advantages, sometimes even beating hierarchical clustering.

3. Compounding the issue, these datasets are used in the Appendix for deriving default values for key IMPACC parameters, such

Table 1: **Data sets used in empirical study.**

	**PANCAN**	**Biase**	**Goolam**	**Yan**	**COIL20**
**Data type**	RNA-seq	scRNA-seq	scRNA-seq	scRNA-seq	Image
**Tissue**	tumor cells	mouse embryos	mouse embryos	human embryos	
**# clusters**	5	3	5	7	20
**# observations**	801	49	124	90	1,440
**# features**	20,531	25,737	41,480	20,286	1,024
**% zeros**	14.2%	50.43%	68.56%	38.08%	34.38%
**citation**	Weinstein et al. [2013]	Biase et al. [2014]	Goolam et al. [2016]	Yan et al. [2013]	Nene et al. [1996]
**Source**	Synapse:syn4301332	GSE57249	E-MTAB-3321	GSE36552	

as the observation and feature sample sizes used during the consensus clustering iterations.

We thank the reviewer for this suggestion, and we conducted new hyper-parameter tests on all our real data sets, including the PANCAN bulk-cell RNA-seq, COIL image data, and the three new benchmark single-cell RNA-seq data sets. We found that our methods are fairly robust to the changes of hyperparameters, and our empirical analysis demonstrates that we can achieve promising results in both synthetic data and real data sets using the default hyper-parameters. Additionally, we offer a hyper-parameter tuning procedure in a data-driven fashion using the confusion value as a metric, as discussed in Appendix H.

4. There is also lack of adequate detail to properly assess some of the conclusions drawn. E.g., in Table 3, KEGG pathway enrichment analysis results are presented demonstrating that pathways and enrichment scores acquired by using the top gene features identified by IMPACC are more informative than what is gotten when using the (significantly fewer) top genes from sparseKM and sparseHC. But this may just be a consequence of how the other tools were run. E.g., using a small lasso penalty for sparseKM will constrain the algorithm to select a small set of features; this set can still lead to accurate clustering but will be devoid of highly correlated genes, i.e., precisely the genes that would be beneficial in enrichment analysis. This is not necessarily a limitation. In practice, the typical approach is to follow clustering with differential gene expression analysis, to identify genes that are differentially expressed in a given cluster, and then use those genes for pathway analysis.

We thank the reviewer for this suggestion. As shown in the response to the 4th point of Reviewer 1, we revised the biological interpretation part and conducted a more solid analysis of the top features selected by IMPACC in the Yan[Yan et al., 2013] data set. We also propose a data-driven way to set the cutoff as the mean plus one standard deviation of all scores. Taking the Yan[Yan et al., 2013] data set as an example, IMPACC selects 466 differentially expressed genes using the data-driven cutoff, and the full list of genes can be found in Table S2. We show that the important genes selected by IMPACC are actually differentially expressed genes, as shown in Figure 1. The impor-

tant genes selected by IMPACC have significantly different expressions among different clusters of cells, especially in the Morulae cluster.

Then we conducted GO enrichment analysis on the selected differentially expressed genes, and confirmed that IMPACC is able to provide accurate and reliable interpretations of scientifically important genes as well as biologically meaningful GO enrichment analysis; these results match the original paper's scientific conclusions in which the cell types are known. And the complete list of GO terms and pathway analysis using differentially expressed genes identified by SC3 and sparseKM can be found in Appendix E and Table S3.

5. Finally, attention needs to be paid to the overall quality of the manuscript. Variables in pseudocodes are used without definition. In most cases, the definition is hidden in the Appendix. But there are also variables that are just never defined (e.g., "E" in Algorithm 3). In some places indices appear to be missing (e.g., "u" in the nominator of the expression in step 2, of Algorithm 2). There are occasional discrepancies between the text and the pseudo code. E.g., the text states "(t) [0.5, 1] is a monotonically increasing parameter that controls sampling size". But the value (t) appears constant in the code for Algorithm 3. And almost uniformly, initialization values for variables are not provided.

We thank the reviewer for this suggestion. We have revised the manuscript thoroughly and revised the corresponding details. To clarify, $\{\{\gamma^{(t)}\} \in [0.5, 1], t = 1, 2, ..\}$ is a monotonically increasing sequence, and the values $\gamma^{(t)}$ at a specific t would be a constant.

6. Data presented in Figure 15 of the Appendix, raise some questions. Specifically, for a given selection of n (size of feature sample), one would expect that clustering performance would monotonically improve with an increase in m (size of observation sample). This is clearly not the case for the PANCAN dataset, where, e.g., for n =-0.3, there is a precipitous drop in ARI from almost 1 (m = 0.2) to 0.75 (m = 0.5). Why is this?

We thank the reviewer for this question. It actually makes sense to have a drop in clustering accuracy with more observations in a minipatch. A minipatch with more samples can be a more difficult clustering task since it contains additional uncertain observations. Specifically, we are likely to have more observations lie on the cluster boundaries, which would hamper clustering accuracy and stability.

7. Feature weighting in Algorithm 4 uses simple ANOVA, without considering feature correlation/collinearity. Given the highly correlated nature of gene expression, this approach could up-weight highly correlated features at the expense of other possibly informative features. The synthetic dataset, as constructed, would not be able to surface this limitation due to its artificially over-constrained feature correlation structure.

We thank the reviewer for this suggestion. As shown in the response to the point 2 of reviewer 1, we explore alternatives on how to evaluate feature impor-

tance with a minipatch. Our current IMPACC evaluates the level of association between each feature and the estimated cluster labels on that minipatch by a one-way ANOVA test. We agree with reviewer 2 that the ANOVA test does not consider feature collinearity. So we further propose to conduct multinomial regression of feature values to cluster assignment, which can take the feature correlation into account. Specifically, one feature's importance score is calculated as the mean of predicted probabilities on the cluster assignments from the fitted multinomial regression.

As shown in Figure 1, IMPACC variants that implement rank ANOVA test or multinomial regression do not demonstrate significant advantages in clustering accuracy, measured by ARI. Also, the variants have much more significant computational burdens than the IMPACC with a simple ANOVA test.

8. For the comparisons against the 3rd party clustering algorithms, details of precisely how these algorithms were executed need to provided (e.g., in the Appendix). Otherwise it is not possible to assess if these analyses were performed properly. For instance, for the neoplastic dataset, the authors report a rather dismal ARI of 0.346 for the very clustering method (tSNE + kMeans) used in the publication introducing and analyzing this dataset (PMID: 29091775) and which, presumably, generated the cluster labels. This is highly surprising.

We thank the reviewer for this suggestion, and we add details on the execution of competing methods in Appendix C.

- IMPACC and MPCC: we use default hyper-parameters and apply the early-stopping rule;
- Consensus clustering: the number of observations to subsample and number of iterations is set to be the same as MPCC with an early-stopping rule;
- sparse KMeans: we first tune hyper-parameters with the provided permutation function, setting the range of parameters [3,7]; then, we apply sparse KMeans clustering with the selected optimal hyper-parameter and oracle number of clusters.
- sparse hierarchical clustering: we first tune hyper-parameters with the provided permutation function, setting the range of parameters to be [1.5,6]; then we apply sparse hierarchical clustering with the selected optimal hyper-parameter and average linkage;
- Seurat: we conduct the following steps to perform Seurat clustering:
    1. Using the count data without log transformation, we first find a subset of 2000 features that exhibit high cell-to-cell variation using *vst* method;
    2. Then, we scale the selected subset of data and apply PCA dimension reduction;

3. Using the first 50 PCs, we group the observations with the resolution value that can result in the oracle number K of clusters.

Note that we only apply Seurat to single-cell data sets.

- SC3: we first create a SingleCellExperiment object with count and log-transformed data; then perform SC3 with the number of clusters being oracle and other hyper-parameters being the default. Note that we only apply SC3 to single-cell data sets;
- KMeans: we apply KMeans to the scaled and log-transformed data with oracle number of clusters;
- Hierarchical clustering: we apply hierarchical clustering to the scaled and log-transformed data with oracle number of clusters, using ward.D linkage with Manhattan distance;
- Spectral clustering: we apply spectral clustering to the scaled and log-transformed data with oracle number of clusters, using rbfdot kernel;
- KMedoid clustering: we apply KMedoid clustering to the scaled and log-transformed data with the oracle number of clusters;
- Clusterings after t-SNE dimension reduction: we first apply t-SNE dimension reduction to the scaled and log-transformed data, with perplexity being 30; then we apply KMeans/ Hierarchical clustering/ Spectral clustering/ KMedoid clustering to the reduced dimensions, with settings the same as above.

9. The real data sets used should be described in more detail. The authors should also clarify the statement "We apply our methods to three RNA-seq data sets and one image data set with known cluster labels", and specifically what is the source of the "known cluster labels". The details in Table 1 should also be double checked for accuracy. E.g., the GEO accession number provided for the PANCAN data set is likely wrong. Similarly, for the other GEO datasets, the number of observations listed in Table 1 is different from the number of samples listed in GEO for the indicated GSE ids. The source of the discrepancy should be explained.

We thank the reviewer for this suggestion. We removed the study on our current single-cell data sets and added three new gold standard single-cell data sets: Biase[Biase et al., 2014], Goolam[Goolam et al., 2016], and Yan[Yan et al., 2013], which are also studied in the SC3 paper [Kiselev et al., 2017]. The cluster information of the three single-cell RNA-seq data is known because these data sets are generated from cells of various development stages. The Biase[Biase et al., 2014] data is generated from 49 single cells composed of 1-cell (zygote), mid-stage 2-cell, and 4-cell mouse embryos. The Goolam[Goolam et al., 2016] data investigates gene expression patterns in the pre-implantation development

of mouse embryos, including cells isolated from the 2-cell stage to 32-cell stage. And the Yan[Yan et al., 2013] data measures gene expression of cells from human preimplantation embryos and human embryonic stem cells at different passages. Details are listed in Table 1. The PANCAN bulk-cell RNA-seq data set measures the expression of different tumor cells. We summarize the mean of 10 realizations of clustering results on real data sets. We show that IMPACC is either the best or among the top performing methods in each data set at discovering known clusters with high ARI scores. And IMPACC demonstrates major computational advantages, sometimes even beating hierarchical clustering.

10. The clustering problem statements reads: "We assume that the observations can be separated into K non-overlapping and exhaustive clusters; our goal is to find these clusters." This is too informal. Any set of $>=$ K observations can be separated arbitrarily into K non-overlapping and exhaustive clusters. More precise language should be used, e.g., recasting the problem in optimization terms.

We thank the reviewer for this suggestion, and we have revised the expression.

4 Reply to reviewer 3

1. The authors provided the codes for the implementation of the described methods. However, the documentation for the usage of those codes is lacking, which may make it difficult for other people to use this tool. The authors are encouraged to organize those individual R scripts into an R package (ideally a Bioconductor package) with proper documentation (function help page, tutorial, example and so on.)

We thank the reviewer for this suggestion. We made the IMPACC package available on GitHub at `https://github.com/DataSlingers/IMPACC`, and we are sending it to Bioconductor.

2. The authors used way too many "dramatic" in this manuscript. In my opinion, it's better to refrain from using such words, especially when the improvement of computational efficiency and accuracy in some scenarios is rather comparable to some other clustering methods and the performance also depends on whether the dataset itself is sparse or not, as mentioned by the authors in the discussion part.

We thank the reviewer for this suggestion. We have revised the related expression in the manuscript.

3. The authors are encouraged to strengthen the hyper-parameter tuning part and better justify the generalizability of the recommended hyper-parameters. The authors showed that hyper-parameters had a limited impact on model performance and therefore suggested that users could choose the default parameters. However, the authors only tuned and compared the hyper-parameters on two real-world biological datasets (Brain cells and PANCAN). As the users will perhaps have much more diverse biological datasets, the authors may want to test the hyper-parameters in more cases (other types of biological data or synthetic data) to ensure the generalizability of the recommended hyper-parameters.

We thank the reviewer for this suggestion. As illustrated in the response to the point 3 of reviewer 2, we conducted new hyper-parameter tests on all our real data sets, including the PANCAN bulk-cell RNA-seq, COIL image data, and the three new benchmark single-cell RNA-seq data sets. We found that our methods are relatively robust to the changes of hyper-parameters, and our empirical analysis demonstrates that we can achieve promising results in both synthetic data and real data sets using the default hyper-parameters. Additionally, we offer a hyper-parameter tuning procedure in a data-driven fashion using the confusion value as a metric, as discussed in Appendix H.

References

Fernando H Biase, Xiaoyi Cao, and Sheng Zhong. Cell fate inclination within 2-cell and 4-cell mouse embryos revealed by single-cell rna sequencing. *Genome*

research, 24(11):1787–1796, 2014.

Mubeen Goolam, Antonio Scialdone, Sarah JL Graham, Iain C Macaulay, Agnieszka Jedrusik, Anna Hupalowska, Thierry Voet, John C Marioni, and Magdalena Zernicka-Goetz. Heterogeneity in oct4 and sox2 targets biases cell fate in 4-cell mouse embryos. *Cell*, 165(1):61–74, 2016.

Vladimir Yu Kiselev, Kristina Kirschner, Michael T Schaub, Tallulah Andrews, Andrew Yiu, Tamir Chandra, Kedar N Natarajan, Wolf Reik, Mauricio Barahona, Anthony R Green, et al. Sc3: consensus clustering of single-cell rna-seq data. *Nature methods*, 14(5):483–486, 2017.

Sameer A. Nene, Shree K. Nayar, and Hiroshi Murase. Columbia object image library (coil-20). Technical report, 1996.

John N Weinstein, Eric A Collisson, Gordon B Mills, Kenna R Mills Shaw, Brad A Ozenberger, Kyle Ellrott, Ilya Shmulevich, Chris Sander, and Joshua M Stuart. The cancer genome atlas pan-cancer analysis project. *Nature genetics*, 45(10):1113–1120, 2013.

Daniela M Witten and Robert Tibshirani. A framework for feature selection in clustering. *Journal of the American Statistical Association*, 105(490):713–726, 2010.

Liying Yan, Mingyu Yang, Hongshan Guo, Lu Yang, Jun Wu, Rong Li, Ping Liu, Ying Lian, Xiaoying Zheng, Jie Yan, et al. Single-cell rna-seq profiling of human preimplantation embryos and embryonic stem cells. *Nature structural & molecular biology*, 20(9):1131–1139, 2013.

Luke Zappia, Belinda Phipson, and Alicia Oshlack. Splatter: simulation of single-cell rna sequencing data. *Genome biology*, 18(1):1–15, 2017.
