# Supplementary material for: Fast and interpretable consensus clustering via minipatch learning
Source: PLoS Comput Biol. 2022 Oct 3;18(10):e1010577. doi: 10.1371/journal.pcbi.1010577 (PMC9560608; doi:10.1371/journal.pcbi.1010577)
Supplement: Attachment — Submitted filename: reply2.pdf [file pcbi.1010577.s006.pdf]

Response to reviews for PCOMPBIOL-D-21-02086: Fast and Interpretable Consensus Clustering via Minipatch Learning

July 24, 2022

1 Response to Reviewer and AE Comments

We thank the reviewers and the action editor for their careful reading and thoughtful feedback. We have made several changes to the paper in response to these comments and believe the paper is significantly stronger. The main changes in the manuscript are all marked in blue for clarity and we provide our detailed response to the reviewers' comments in the following sections.

2 Reply to Reviewer 1

Figure 3 of the revised manuscript shows the differentially expressed genes that they identify through their methods and figure 4 shows the relevant pathways. These mainly include genes/pathways down-regulated as development progresses. However, in the main manuscript they argue that they "successfully identify 26 GO terms, and these pathways are highly related to the regulation of the reproductive process and cell development" (page 15; line 418). In addition they mention that in the original publication Yan et al. "discovered that the differential genes between EPI cells and the remaining cells are enriched for GO terms related to transcriptional regulation and germ cell development". Why is germ cell development (e.g. oogenesis and oocyte differentiation) relevant when the biological context is pre-implantation development? I think the authors need to better clarify the parts of biology that their methodology captures. One way of doing so would be to better argue on the importance of these pathways in this context (it makes sense biologically for the negative regulators of sperm binding to the zona pellucida to be downregulated after proper fertilization) but I would expect a much more thorough discussion. Another way is to show some Venn diagrams of the identified pathways with pathways relevant in mouse pre-implantation development. This is a well-studied period of development with many datasets and results publicly available that the authors can utilize and justify the robustness of their results or to better showcase their own differential expression analysis.

We thank the review for the question. To clarify, the study of Yan et al is conducted on "transcriptome of human oocytes and early embryos at seven developmental stages—from metaphase II mature oocytes to late blastocysts—and of the primary outgrowth during hESC derivation (hESCs of passage 0) and hESCs of passage 10", and the goal of this study is to "address the long-standing question whether gene expression signatures of human epiblast (EPI) and in vitro hescs are the same". Then Yan et al. are able to find "3,906 genes showed *differential expression between EPI and the remaining cells.*" , which are enriched for "GO terms related to transcriptional regulation and germ cell development, indicating that EPI cells have lower expression of genes related to gamete generation, germ cell development and reproduction compared to the other cell lineages in blastocysts". Therefore, the pathways identified by differentially expressed genes in this study reflect the difference between EPI cells and blastocysts cells.

We are able to identify similar GO pathways with IMPACC, related to germ cell development (oogenesis, oocyte development, oocyte differentiation, egg coat, structural constituent of egg coat, etc.), gamete generation(female gamete generation, female gamete generation, etc.), and reproduction (regulation of reproductive process, negative regulation of reproductive process, positive regulation of reproductive process, cellular process involved in reproduction in mul-

ticellular organism, etc.), as shown in Appendix Table 1. In addition, sparseKM can also identify cell development related GO terms (oogenesis, oocyte development, oocyte differentiation, egg coat, structural constituent of egg coat), as shown in Appendix Table 2. We clarified the confusion in the manuscript and added a Venn diagrams of pathways identified by IMPACC and SparseKM in Appendix.

3 Reply to Reviewer 2

1. "Here we only show the results of sparse simulation with autoregressive covariance structure, as it is the best representative of high dimensional bioinformatics data." Justification for this statement ("best representative of high dimensional bioinformatics data") should be provided, e.g., via citing work where this is demonstrated. And a rationale for the specific choice of covariance (j,j = |jj|) should be given.

We thank the reviewer for this suggestion. We rephrased the corresponding statement in our manuscript.

2. In my opinion, the results of clustering the Splatter-simulated data are more reflective of the algorithm's performance on real scRNA-seq data and, in fairness, should at the very least be presented in the main text along with the results from the autoregressive model, rather than in the appendix.

We thank the reviewer for this suggestion and we do think the splatter simulation is a very relevant study. However, we believe that the auto-regressive simulation is much more effective in showing the differences among various methods, while it is difficult to illustrate the comparable differences in splatter simulation. Therefore, we put the splatter simulation into supplementary materials.

3. One recommendation for Table 2: in addition to displaying the actual measurements, consider using color gradients for the table cells (similar to how you present data in Appendix Figure 21), to aid visual delineation of "good" and "bad" values.

We thank the reviewer for this recommendation. We use bold font for the highest ARI in each method. However, we think that the color gradients in this table may be misleading as the ARI and Time values are at different scales.

4. "Clustering followed by dimension reduction via tSNE can have faster and better clustering accuracy for some of the data sets, but they fail to provide interpretability in terms of feature importance". I disagree with the authors that this is a significant limitation. E.g., one can always run a post-clustering ANOVA (or similar) to prioritize features, if desired.

We thank the reviewer for this question. We agree that tSNE+post-clustering ANOVA is a widely used method. However, it is a two-step method and the post-selection results are not statistically correct. Zhang et al. [2019] shows that clustering and running DE analysis on the same dataset would lead to selection

bias in single-cell RNA-seq. And Berk et al. [2013], Fithian et al. [2014] proved clustering could lead to false discoveries. We also added these citations in the manuscript. Besides, the method we proposed is a one-step method and there is no need for any post-selection process.

References

Richard Berk, Lawrence Brown, Andreas Buja, Kai Zhang, and Linda Zhao. Valid post-selection inference. *The Annals of Statistics*, pages 802–837, 2013.

William Fithian, Dennis Sun, and Jonathan Taylor. Optimal inference after model selection. *arXiv preprint arXiv:1410.2597*, 2014.

Jesse M Zhang, Govinda M Kamath, and N Tse David. Valid post-clustering differential analysis for single-cell rna-seq. *Cell systems*, 9(4):383–392, 2019.
